# Short- and Medium-Chain Chlorinated Paraffins in Polyvinylchloride and Rubber Consumer Products and Toys Purchased on the Belgian Market

**DOI:** 10.3390/ijerph18031069

**Published:** 2021-01-26

**Authors:** Thomas J. McGrath, Giulia Poma, Hidenori Matsukami, Govindan Malarvannan, Natsuko Kajiwara, Adrian Covaci

**Affiliations:** 1 Toxicological Centre, University of Antwerp, Universiteitsplein 1, 2610 Wilrijk, Belgium; giulia.poma@uantwerpen.be (G.P.); malarvannan.govindan@uantwerpen.be (G.M.); 2 Center for Material Cycles and Waste Management Research, National Institute for Environmental Studies (NIES), 16-2 Onogawa, Tsukuba 305-8506, Japan; matsukami.hidenori@nies.go.jp (H.M.); kajiwara.natsuko@nies.go.jp (N.K.)

**Keywords:** chlorinated paraffins, SCCPs, MCCPs, consumer products, polyvinyl chloride, rubber

## Abstract

This study investigates the presence of Stockholm Convention listed short-chain chlorinated paraffins (SCCPs) and their replacement medium-chain chlorinated paraffins (MCCPs) counterparts in polyvinyl chloride and rubber consumer products and toys purchased on the Belgian market in 2019. SCCPs were detected in 27/28 samples at concentrations ranging from <LOQ–130,000 µg/g with a median level of 2.5 µg/g, while MCCPs were detected in only five samples ranging <LOQ–3500 µg/g. Levels of SCCPs in all but one of the samples were below the European Union’s guideline limit of 0.15%, by weight, and concentrations of both SCCPs and MCCPs in the majority of products suggested unintentional incorporation to the polymeric materials. The homologue distribution of SCCPs was generally dissimilar to known commercial formulations and appeared to be indicative of contamination during manufacture or via recycling of previously treated goods. MCCP patterns, conversely, were broadly representative of those reported for industrial mixtures and may have been inadvertently incorporated via the application of mixed carbon-chain length CP formulations or recycled goods. This research suggests that overall SCCP presence has decreased in goods on the European market compared with previous reports and that both SCCPs and MCCPs may still enter EU marketplaces from unintentional sources.

## 1. Introduction

Chlorinated paraffins (CPs) are industrial chemicals used in a broad array of applications as flame-retardants and plasticizers. CPs consist of chlorinated *n*-alkanes ranging from C_6_ to C_38_ which are commonly categorized as short-chain (SCCPs; C_10_–C_13_), medium-chain (MCCPs; C_14_–C_17_) and long-chain (LCCPs; C_>17_) [1] with weight-based chlorination degrees ranging from approximately 30–70%. Concerns regarding the environmental persistence, bioaccumulation potential and toxicity of SCCPs [2,3,4] has attracted considerable research attention in recent years, culminating in registration as Stockholm Convention persistent organic pollutants (POPs) in 2017 [5]. Despite indications that MCCPs and LCCPs may share many of the harmful characteristics of SCCPs, these homologue groups appear to have been implemented widely as replacements for the banned substances [6,7,8,9,10].

Large-scale production of CPs began in the 1930s with global production increasing from the 1970s [6,11] when manufacture began in Japan and European countries like the UK and Germany [4,6,12]. Production expanded again dramatically after 2006 as Chinese manufacturing rose from 260,000 tonnes/year (t/y) to more than 1,000,000 t/y in 2013 [13]. Overall, CP production was estimated to comprise of approximately 16.5% SCCPs in 2013 although proportions are expected to have declined in recent years owing to SCCP POPs status [6,13]. Less information is available regarding the relative fractions of MCCPs and LCCPs in the current market and assessments are complicated by the fact that many manufacturers do not differentiate CP formulations by carbon-chain length [10].

The diverse physicochemical properties of CPs afforded by their range of carbon-chain lengths and chlorination degrees make them especially versatile. CPs have been utilized for their fire-retarding and plasticizing attributes in metal-working fluids, lubricants, polymers, paints, coatings, sealants, adhesives, leather and textiles [3]. Application in polyvinyl chloride (PVC) as a plasticizer and flame retardant has been reported among the major uses of SCCPs [1,3]. SCCPs may provide beneficial properties to synthetic materials at concentrations of 1–4% [11] but can be added to PVC at levels of up to 10% weight/weight (*w*/*w*) [13]. A large proportion of PVC is used for wall and floor coverings, upholstery and electrical cable insulation, while its soft and flexible qualities are also suitable for personal consumer items such as toys, sporting equipment and footwear [3]. Another of the most common CP uses is in synthetic rubber formulations (up to 17% *w*/*w*) [13], which are prevalent as construction materials, industrial conveyor belts and in many consumer products [12]. Indeed, levels of SCCPs and MCCPs were significantly higher in Chinese PVC and rubber goods than in items made from polyethylene terephthalate, polypropylene or polyethylene [14], and both CP homologue groups have been detected in a range of products purchased on the European market [3,7,9,15,16].

Release of SCCPs via volatilization has been evidenced from PVC flooring during thermal treatment by Zhan et al. [17] while SCCPs, MCCPs and LCCPs have been observed to migrate from other treated materials [9,15,18]. Such leaching from CP-treated products can lead to contamination of environmental matrices and indoor dust, resulting in human exposure [10,19,20]. For items such as toys or sporting equipment, exposure via direct dermal contact or mouthing by children and toddlers must also be considered. Evidence of CP toxicity and environmental persistence has been mounting in recent years [4,10]. SCCPs have been shown to exhibit neurotoxic and endocrine disrupting properties [21,22,23] and have been classified as potential carcinogens in the EU since 2008 [24], while MCCPs have been studied less widely but have indicated similar toxic behavior in some studies [10,25]. SCCPs have accordingly faced restrictions on use and manufacture in the European Union (EU) and elsewhere since the early 2000s [12]. Directive 2002/45/EC [26] restricted the allowable levels of SCCPs in substances used metal-working and fat liquoring for leather to 1% and limits for other articles produced or placed on the market in the EU were later set to 0.15% by weight by Directive 2015/2030 [27]. SCCP registration as POPs under Annex A of the Stockholm Convention stipulates a limit of 1% of SCCPs by weight in CP mixtures and also includes several usage exemptions [5,28]. Various legislated and voluntary restrictions on SCCPs have been implemented in other countries including the USA, Canada, Japan, Norway and China [3,12,29]. A lack of unified global approach to restriction of SCCPs in manufactured goods, however, creates the potential for transboundary importation of treated products to markets in which SCCPs are more heavily regulated. To the authors’ knowledge, MCCP and LCCP formulations remain unrestricted globally.

To date, analyses of CPs in polymeric materials have largely focused on materials used for purposes other than personal consumer items, and few assessments have been published [13]. This study aims to determine the levels of SCCPs and MCCPs in PVC and rubber consumer products and toys purchased on the Belgian market to evaluate the current status of these chemicals concerning current EU limitations on production and import of treated products. An interrogation of congener group patterns in the analyzed goods was undertaken to further elucidate potential sources of CPs to PVC and rubber items to aid future endeavors in reducing the presence of harmful substances in the EU marketplace.

## 2. Materials and Methods

### 2.1. Standards and Reagents

Technical mixture standards of SCCPs (55.5 and 63% Cl) and MCCPs (42 and 57% Cl) were purchased from Dr Ehrenstorfer GmbH (Augsburg, Germany). Analytical standards of plasticizers di-2-ethylhexyl-terephthalate (DEHT), 1,2-cyclohexane dicarboxylic acid diisononyl ester (DINCH), phthalic acid esters di-n-butyl phthalate (DNBP), bis-2-ethylhexyl phthalate (DEHP) and di-(2-propyl heptyl) phthalate (DPHP) were purchased from AccuStandard (New Heaven, CT, USA). LC/MS grade methanol, Japan Industrial Standard grade ammonium acetate and dioxin-analysis-grade toluene, *n*-hexane, acetone and silica gel impregnated with 55% *w*/*w* sulphuric acid were obtained from Fujifilm Wako Pure Chemical Corporation (Osaka, Japan). Dioxin-analysis-grade dichloromethane was purchased from Kanto Chemical Co. Inc. (Tokyo, Japan) and Milli-Q water was supplied by a Synergy UV ultrapure purification system (Millipore Corporation, Billerica, MA, USA). Pesticide grade *iso*-octane was purchased from VWR (Leuven, Belgium).

### 2.2. Sampling and Sample Preparation

Twenty-eight consumer product samples representing common household goods, including children’s toys, exercise equipment, footwear and electrical cables were purchased on the Belgian market in June 2019. Products composed of PVC (*n* = 19) and rubber (*n* = 6) were selected due to the reportedly common application of CPs in these polymers [1,3,13] (Table 1). Sample materials were identified by packaging labels (Supplementary Materials Section S1), although the composition of three samples could not be determined. Purchased items were manufactured in several Asian and European countries, allowing investigation of potential CP application differences by geographical location. The year of manufacture was not available.

Subsections of each sample were cut into small pieces (about 1 cm × 1 cm) using acetone-rinsed stainless-steel scissors and then pulverized by a freezer mill to a fine powder. From each sample homogenate, 0.2 g was extracted by ultrasonication in 10 mL of toluene for 30 min. Aliquots of 0.5 mL were taken from the toluene extracts, diluted in 3 mL of dichloromethane:*n*-hexane (1:9 *v*/*v*) to precipitate polymer matrix components, combined with 1 g of acidified silica (55% *w*/*w*) and vortexed for 30 s. The supernatant was collected, evaporated to 0.1 mL and then redissolved in 1 mL of methanol for analysis by liquid chromatography–tandem mass spectrometry (LC–MS/MS). An aliquot of 0.1 mL of the final extract was evaporated to dryness and reconstituted in *iso*-octane for analysis by gas chromatography–mass spectrometry (GC–MS).

### 2.3. LC–MS/MS Analysis and Quantification

LC–MS/MS analysis was performed on a Waters ACQUITY UPLC H-Class/Xevo TQ-S microsystem operated in the electrospray ionization (ESI) negative mode as described by Matsukami et al. [30]. Sample injections of 5 µL were separated using an Agilent ZORBAX SB-CN column (100 mm × 2.1 mm, i.d. 1.8 μm) at 40 °C. Mobile phase A consisted of Milli-Q water containing 5 mM of ammonium acetate and mobile phase B was methanol with 5 mM of ammonium acetate. The flow gradient was 0 min (60% B), 15 min (99% B), 17 min (99% B) and 17.1 min (60% B) at a constant flow rate of 0.4 mL/min. The nitrogen desolvation gas temperature was 500 °C at a flow rate of 1000 L/h. The capillary voltage was 0.75 kV, the source temperature 110 °C and argon was the collision gas. Two mass transitions were monitored for each congener group, which were considered detected when the signal to noise ratio (S/N) was greater than 3, retention time was within ±5% of peaks in standards and the ratio between the first and second transitions within ±20% of standards (Supplementary Materials Table S1). Congener groups were quantified when S/N > 10 using individual calibration curves constructed from technical mixtures of SCCP 55.5 and 63%Cl in 1:1 ratio (0.1–20 µg/mL) and MCCP 42 and 57%Cl in a 1:1 ratio (0.01–10 µg/mL). Congener percentage compositions in technical mixtures were determined using the amount of stable isotopes of [M−Cl−2HCl]^+^ ions obtained by GC–high resolution (HR) MS analysis according to the calibration standard procedure described for SCCPs by Matsukami et al. [30]. Additional analytical details with respect to GC–HRMS parameters for the measurement of MCCP congeners are given in the Supplementary Materials Section S2 and Table S2. Congener compositions of SCCP and MCCP technical mixtures are shown in Supplementary Materials Tables S3 and S4, respectively.

### 2.4. GC–MS Analysis and Identification of Other Plasticizers

Sample extracts were also analyzed using an Agilent 6890 GC coupled to a 5973 MS operated in electron impact (EI) ionization mode to investigate the presence of plasticizers other than CPs. Pulsed splitless injections of 1 µL were delivered to the inlet at a temperature of 300 °C and an HT-8 capillary column (25 m, 0.22 mm internal diameter and 0.25 µm film thickness) was used to separate analytes at a constant helium flow rate of 1 mL/min. The oven temperature program entailed an initial hold of 3 min at 60 °C followed by a ramp of 12 °C/min to 300 °C and isothermal hold at 300 °C for 15 min. The MS was operated in scan mode from 40 to 800 *m*/*z* with the ion source and quadrupole at 230 °C and 150 °C, respectively. Analytes were identified by comparing mass spectra and retention times to analytical standards and the National Institute of Standards and Technology (NIST) mass spectral library [31].

### 2.5. Quality Assurance and Quality Control

Extraction experiments by ultrasonication in both toluene and tetrahydrofuran were conducted using four samples of polyvinyl chloride containing SCCPs and MCCPs (Supplementary Materials Table S5). Based on the results obtained from the extraction experiments, toluene was used as an appropriate extraction solvent in this study. The instrumental detection limit values for the individual congeners of SCCPs and MCCPs ranged from 2 to 100 pg, and the limit of quantification (LOQ) values for the individual congeners in polyvinyl chloride samples ranged from 0.001 to 0.01 µg/g. The concentrations of analytes in procedural blanks were below the LOQ values in this study.

### 2.6. Statistical Analyses

Descriptive statistics were calculated using Microsoft Excel version 16.

## 3. Results and Discussion

### 3.1. SCCP Concentrations in Consumer Goods

Summary concentrations of ∑SCCPs and ∑MCCPs in consumer products and toys samples are presented in Table 2 and the complete dataset is detailed in Supplementary Materials Tables S6 and S7. SCCPs were identified in 96% of samples with overall ∑SCCPs concentrations ranging from <LOQ to 130,000 µg/g and a median of 2.5 µg/g. A slightly higher median level of ∑SCCPs was recorded in rubber products, 4.5 µg/g (range; <LOQ–8.0 µg/g) than that of PVC items, 2.2 µg/g (range; 1.3–82 µg/g), although three samples of unknown composition could not be included in this comparison. The very high maximum concentration of 130,000 µg/g (13% *w*/*w*) ∑SCCPs was observed in sample CL, a polymer coated wire clothesline, which was the only item to breach the EC [27] Regulation 2015/2030 limit of 0.15% SCCPs in articles produced or placed on the market within EU. The country of manufacture for this sample, China, introduced limitations on SCCPs in the production of synthetic running tracks in November 2018 (standard GB 36246-2018, maximum 0.15% SCCPs) [29], but to the authors’ knowledge, it does not regulate SCCPs in other consumer products. Since the year of manufacture is unknown for this sample, it is also possible that this item was imported to the EU prior to the implementation of current SCCP limits. Unfortunately, the material makeup of the clothesline sample could not be determined from package labeling. Synthetic wire coatings of electrical cables have been shown to contain high SCCP levels in a range of samples collected on the European market between 2013–2017 [3]. ∑SCCP concentrations in the cables of 15 products like electric shavers, speakers and digital thermometers ranged from 1100 to 47,000 µg/g with a median of 10,000 µg/g. This equates to 0.11–4.7% *w*/*w* such that all but one of the samples in the UNEP report breach the Regulation 2015/2030 value. Cable sheaths purchased in China (n = 11) contained concentrations, which were higher still, ranging from 250 to 190,000 µg/g (0.025–19% *w*/*w*) with a mean level of 84,000 µg/g (8.4% *w*/*w*), although two electrical cables analyzed in the study contained only trace levels of SCCPs [14].

With the exception of sample CL, levels of ∑SCCPs in the other samples spanned a similar range, each in the low µg/g region, up to 82 µg/g. These concentrations represent proportional contributions to the PVC and rubber polymers in the range of approximately 0.0001–0.01% *w*/*w*. This suggests that the SCCP levels present in most of the products analyzed in the current study did not result from intentional application for plasticizing or flame-retardant properties. Polymers may become contaminated with lower levels of SCCPs during production from treated manufacturing components such as conveyor belts or mechanical lubricants, each of which falls within specific exemptions for SCCP application under the Stockholm Convention [27,28]. Conveyor belts have been shown to contain SCCPs [14] that could be released via particulate wear during operation [32], while CP leaching from electric motors and plastic coatings has been evidenced experimentally [9]. SCCPs may also become incorporated into new consumer goods inadvertently via the recycling of previously treated materials. For example, Brandsma et al. [7] reported evidence that low levels of CPs detected in rubber playground tiles and sporting field granules derived from the recycled car tire feedstocks rather than a purposeful application or accidental contamination during manufacturing. Under the current EU regulations, MCCP and LCCP formulations still in use are allowed to contain SCCP impurities up to 1% [27], which could provide yet another potential source for the low SCCP levels observed in samples.

Few studies have reported on CP levels in consumer goods, globally. A United Nations report on waste management of SCCPs showed all of 96 consumer products purchased in the EU between 2013 and 2017 to contain SCCPs at levels ranging 710–100,000 µg/g [3]. The data included measurements from many of the same product types as this work, such as yoga mats (*n* = 7, 2300–69,000 µg/g), bath/all-purpose mats (*n* = 2, 3600–5300 µg/g), a jump rope (22,000 µg/g) and beach ball (3100 µg/g). The highest ∑SCCP concentrations were recorded in a chicken squeeze toy (100,000 µg/g), sports exercise tube (90,000 µg/g) and plastic toy figure (83,000 µg/g), which were all below that of the clothesline (CL) sample in this study. Overall, the UNEP [3] results showed a much greater SCCP prevalence in goods from 2013 to 2017 than the findings of the present study, which may indicate the efficiency of SCCP usage regulations to influence global manufacturing away from SCCP application in the interceding years.

Other studies have investigated the levels of SCCPs in PVC and rubber used for larger items like construction materials or outdoor running tracks. Rubber granules from sports fields and playground tiles from Europe (mostly the Netherlands) contained ∑SCCPs concentrations of 2.1−9.1 μg/g and 1.9−25 μg/g, respectively, resembling the concentrations in the present study [7]. ∑SCCP levels were much higher in Chinese rubber running tracks (13.8–12,800 µg/g) but typically below 1 µg/g in rubber granules [29]. PVC flooring in China was also reported to contain an average of 2500 µg/g ∑SCCPs (*n* = 4) in one study [14] and 2400 µg/g in another study [17]. Although CP addition to structural materials is informed by different requirements to that of personal-use consumer goods, the presence of SCCPs in such items may provide some insight as to potential sources of SCCPs to products like sporting equipment or toys produced from recycled polymers. While simple and fast screening of SCCP levels remains unviable, SCCP-treated goods will likely continue to enter recycling streams and that importation of consumer products with SCCP concentrations above the EU 0.15% *w*/*w* limitations will be difficult to prevent.

### 3.2. MCCPs Concentrations in Consumer Goods

MCCPs were detected less frequently than SCCPs, identified in only 18% of samples with overall ∑MCCPs concentrations ranging from <LOQ to 3500 µg/g and a median of <LOQ. Of the samples in which MCCPs were present, two samples were composed of rubber (rubber ducks RD-1 and RD-3) and two of PVC (pool mat PM-1 and jump rope JR-2), while one of the products (clothesline CL) was of unknown composition. Each of the samples containing MCCPs was manufactured in countries outside Europe except for one sample (rubber duck RD-3) for which country of origin could not be determined. ∑MCCP levels in the polymer coated wire clothesline (sample CL), 3500 µg/g (0.35% *w*/*w*), were two- to three-orders of magnitude above all other measurements. Given that the SCCP concentration in sample CL was approximately 37 times greater than that of MCCPs in this sample, it is possible that MCCPs were incorporated inadvertently as partial impurities within SCCP technical mixtures [33]. The combined application of SCCPs and MCCPs has been evidenced by Brandsma et al. [7] who found an average ratio of 1:5 SCCPs:MCCPs across a range of rubber products manufactured between 2006 and 2014. The high proportion of MCCPs in samples produced many years before SCCP POPs classification may suggest that MCCPs have been favored for particular applications. Indeed, CPs of different chain length impart slightly different characteristics to polymeric materials, with SCCPs favored for greater fire-retarding capabilities due to higher mass fractional chlorine degrees [3].

Besides sample CL, MCCPs were only detected at low concentrations ranging from 5.8 to 350 µg/g (0.00058 to 0.035% *w*/*w*), which are likely to represent unintentional incorporation into these products. Contamination sources of MCCPs to goods during manufacture are expected to mirror those of SCCPs, since they too have been measured in conveyor belts [14] and mechanical components [9]. Unlike SCCPs, however, usage of MCCPs remains unregulated in the EU and elsewhere so that there is no imperative for manufacturers to avoid application of these compounds directly or as impurities in other plasticizing additives, such as LCCP formulations.

Studies investigating MCCP levels in consumer products purchased in Europe have been limited, particularly for PVC and rubber articles. MCCPs were detected at levels up to 96 ng/cm^2^ in cotton-wipes sampled from the internal components of a 10-year-old oven in Germany, in which SCCPs were not identified [15]. Concentrations were highest in samples from the circuit board housing, exhaust shaft and inside of the oven door, while MCCPs were not detected in wipes from the cable and thermal insulation. Electric blenders purchased in Sweden were also shown to contain MCCPs in various elements, such as self-lubricating bearings and polymer washers [9]. MCCPs were the dominant CP homologue group in car tires, rubber sports fields and rubber granules in the Netherlands, contributing an average of 72% of total CP concentrations in analyses which included SCCPs and LCCPs [7]. Although MCCPs were detected with higher regularity (100%) in the Brandsma et al. [7] analyses, the overall ∑MCCP concentrations reported, ranging from 1.2 to 54 µg/g, were broadly comparable to the levels determined in goods purchased in Belgium. ∑MCCP levels have ranged from 1.3 to 3100 µg/g [14] and 13 to 160,000 µg/g [29] in rubber tracks from Chinese studies to reveal the highly variable concentrations in rubber products. Although no MCCPs were detected in two electrical cables sampled in the present work, ∑MCCP were identified in each of the 11 analyzed PVC cable sheaths from China at a high concentration ranging from 31,000 to 140,000 µg/g [14]. The same study reported the average level of MCCPs in four PVC flooring samples to be approximately an order of magnitude lower, 1800 µg/g.

### 3.3. Congener Group Patterns

C_10_ homologues were the most frequently detected among SCCP groups (93%), followed by C_12_ (32%), C_11_ (21%) and C_13_ (7%). C_10_ homologues were the only SCCPs detected in 16 of the 28 samples and contributed an average of 79% ± 33% to ∑SCCP concentrations. The next most prevalent carbon-chain length group was C_12_, which accounted for an average of 16% ± 30%, followed by C_11_ and C_13_, contributing 4% ± 9% and 1% ± 4%, respectively. Hexachlorinated isomers were the most common among chlorine groups for SCCPs, present in 93% of samples, while all other groups were detected in 25% of samples or less. Cl_6_ congener groups, on average, also contributed the greatest proportion to ∑SCCP concentrations, 68% ± 38%, followed by Cl_5_ groups at 13% ± 26% and low individual proportions ranging from 2–7% for the other Cl levels. The dominance of C_10_ homologues within the consumer goods is in contrast with reported profiles of industrial CP mixtures analyzed by ESI [33] and atmospheric pressure chemical ionization [34,35] ionization techniques. SCCP formulations manufactured in Germany (Witaclor 149) and the UK (Cereclor 60L and Cereclor 70L) mainly comprised of C_13_ homologues and C_12_ groups with much smaller proportions of C_10_ and C_11_ [34]. Similar findings were reported in products CP-42, CP-52 and CP-70 from three different manufacturers in China, although the predominance of C_13_ congener groups was even more pronounced among SCCPs and varying proportions of MCCPs and LCCPs were also described among the different mixtures [33]. Yuan et al.’s [35] analysis of 11 technical products including variants of the Witaclor, Cereclor, Hüls and Hordalub tradenames related a wide variety of homologue distributions, although C_10_ congeners were minor contributors (<7%) to all mixtures. The most prevalent chlorine groups within industrial mixtures are typically Cl_6_ to Cl_9_ and dependent on the overall Cl% of the product as defined by the manufacturer [33,34].

The incongruence between the homologue profiles in Belgian consumer goods and those of technical mixtures supports the hypothesis that SCCPs in many of the samples derive from contamination by the manufacturing equipment rather than trace additions of SCCP formulas via other means. C_10_ CPs form the lightest carbon-chain group analyzed in this study, while hexachlorinated isomers are among the lightest chlorine groups such that these congeners are the most likely to volatilize from manufacturing components. Analyses of SCCP formulations by GC–MS using electron capture negative ionization (ECNI) have shown varying congener group distribution patterns [36,37,38] although the differences in ionization efficiency between GC-ECNI and LC technologies are known to be dependent on carbon and chlorine numbers such that direct comparisons between analyses are not instructive [33,35,39,40].

**Figure 1:**
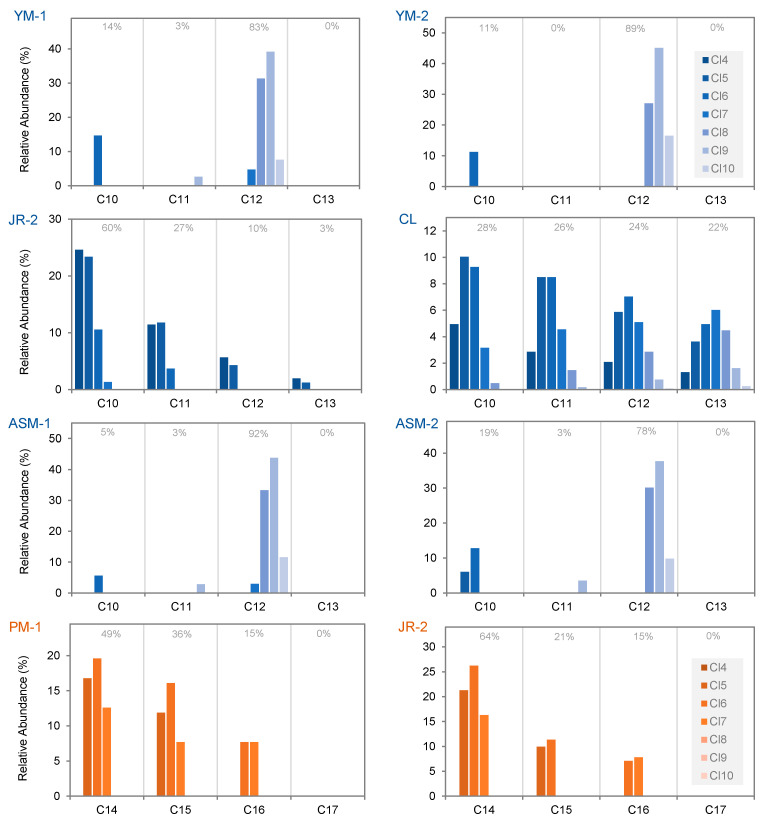

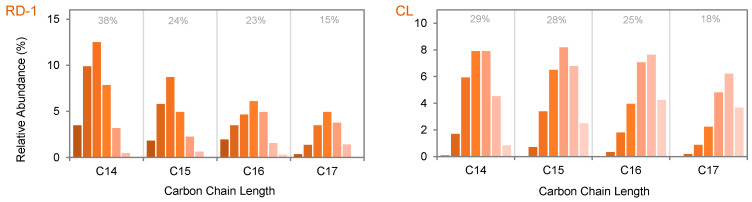
Relative abundance (%) of congener group patterns in the consumer product and toy samples with ∑SCCP (blue) or ∑MCCP (orange) concentrations >10 µg/g. Relative abundance (%) for summed homologues per carbon-chain length is shown above respective carbon-chain length groups.

Congener group patterns of the samples with the highest ∑SCCP concentrations are illustrated in Figure 1. Interestingly, despite being distinct products from separate suppliers and manufactured in at least two different countries, very similar congener group patterns were measured in each of two yoga mats (YM-1 and YM-2) and two antislip mats (ASM-1 and ASM-2). Since the patterns in these four samples, dominated by C_12_ (78–92% relative abundance) and Cl_8_–Cl_10_, do not closely resemble patterns reported in any of the CP formulations to date, the source of these SCCPs is unclear.

Analysis of SCCP mixtures from different suppliers in China has shown large variations in homologue distribution to exist, even between products of the same name (CP-52, for example) [33]. Given that at least 120 CP manufacturers have been identified in China alone [6], it may be possible that these consumer goods have been treated with a formula whose homologue distribution has not been reported in the scientific literature. Congener patterns of sample CL, the only sample containing SCCP concentrations suggestive of intentional application, are also different from the C-chain length patterns reported in individual formulations. This might indicate that more than one CP mixture has been utilized in the manufacture of this item or that the product is composed of a combination of recycled SCCP-treated polymers. Indeed, carbon-chain profiles observed in approximately half of the Chinese rubber track and granule samples measured by Xu et al. [29] resembled those of sample CL, while greater proportions of C_13_ and C_12_ groups were evident in many of the remaining samples from the same study. Brandsma et al. [7] observed SCCP profiles in European rubber tire and granule samples to align with common industrial products, with C_13_ and C_12_ dominant at averages of 40% and 26%, respectively, while rubber and PVC items from China contained more prominent levels of C_11_ and C_13_ congeners [14].

For MCCPs, C_14_ homologues were present in 18% of samples and C_15_, C_16_ and C_17_ groups were detected in 14%, 14% and 7% of samples, respectively. Among the samples in which MCCPs were detected, C_14_ homologues contributed an average of 56% ± 28% of ∑MCCP concentrations while C_15_, C_16_ and C_17_ groups accounted for 22% ± 13%, 16% ± 10% and 7% ± 9%, respectively. Hexachlorinated congener groups were the most prominent in the five samples containing MCCPs, with an average relative abundance of 31% ± 13%, closely followed by hepta- and penta-chlorinated groups, contributing 26% ± 5% and 21% ± 11%, respectively. Smaller proportions of Cl_8_, Cl_9_, Cl_10_ and Cl_4_ averaged 12% ± 12%, 6% ± 11%, 2% ± 5% and 2% ± 3%, respectively. In contrast to the findings for SCCPs, MCCP congener group patterns in consumer goods samples pool mat PM-1, jump rope JR-2 and rubber duck RD-1 were essentially similar to the distribution reported in CP-42 formulations from all three Chinese manufacturers [33] (Figure 1). Comparable patterns were also observed in European rubber samples by Brandsma et al. [7] who reported overall homologue patterns of C_14_ (44%), C_15_ (27%), C_16_ (17%) and C_17_ (12%) in tires, granules and playground tiles. The same order of carbon-chain length prevalence was, likewise, described by Wang et al. [14] for both rubber and PVC materials, although C_14_ dominance was slightly more prominent. The more even pattern of carbon-chain length homologues in sample CL, however, could be indicative of combined application of two or more formulations, such as CP-42 with C_14_ > C_15_ > C_16_ > C_17_ distribution and CP-52 with the inverse C_17_ > C_16_ > C_15_ > C_14_ [33], or recycled source materials.

### 3.4. Presence of Other Plasticizers

Given the low incidence of SCCPs and MCCPs in samples at effective plasticizing levels, sample extracts were analyzed by GC–EI/MS to investigate the presence of other plasticizers in consumer products and toys (Supplementary Materials Table S8). DEHT was the most commonly detected compound, present in 11 out of 19 PVC items (58%) and 4 out of 6 rubber goods (66%). Overall, DINCH, DPHP and DNBP were detected in 5, 2 and 1 samples, respectively, while butyryltributyl citrate (BTBC) was also identified in 3 samples according to a NIST library search. DEHT, DINCH, BTBC and DPHP are often referred to as “alternative plasticizers” (APs) and have become more commonly applied in PVC and other materials since the restriction on the use of some “legacy” phthalates (including DEHP and DNBP and butyl-benzyl-phthalate (BBzP), di-isononyl phthalate (DINP) and di-isodecyl phthalate (DIDP)) were enacted due to health concerns including endocrine disruption and reproductive toxicity [41,42]. DEHP, which was among the most commonly used plasticizers in PVC before restrictions began at the end of the 2000s, was not detected in any samples. The recent restriction of SCCPs in global markets may have partly contributed to a greater uptake of APs to provide plasticizing properties in consumer goods, which could possibly be influenced further by increased scrutiny on MCCPs and LCCPs in the future. Data regarding the toxicity of most APs remains very limited. DEHT and DINCH have been investigated in a small number studies, which suggest lower overall systemic toxicity than DEHP [43,44,45], although research on specific endocrine disrupting and neurodevelopmental endpoints is, at present, lacking [46]. Studies have identified APs in household dust [47] and their respective metabolites in the urine of pregnant women to indicate human exposure [48]. This study illustrates a widespread incidence of these emerging compounds in products and toys on the Belgian market, illustrating potential sources of APs to dust and subsequent human intake. To the authors knowledge this is also the first report of CPs and APs in the same polymers. Further studies are required to determine whether this represents deliberate application of APs to consumer products and toys or contamination during manufacturing sources.

### 3.5. Implications for Human Exposure

The widespread presence of CPs in consumer goods and children’s toys is likely to have implications for human exposure via oral and dermal intake pathways. Of particular concern is the enhanced exposure scenario encountered by children and infants due to mouthing of toys or other items containing CPs. Although the transfer of CPs to saliva has not yet been studied, Wang et al. [18] described strong leaching of SCCPs and MCCPs from food contact materials in a suite of experiments using various food simulants. A median SCCP migration efficiency of 3.9% to water was reported from food packaging of unspecified polymer type (*n* = 6), which had similar concentrations to some of the items of the present study (0.97–4.7 µg/g ∑SCCPs). Although these experiments, conducted at 40 °C for 10 days, do not directly represent the conditions relating to mouthing of PVC and rubber items, this evidence illustrates the potential for migration of CPs from polymers to aqueous matrices, such as saliva.

Other plasticizers and flame retardants have, however, been shown to leach into synthetic saliva via experiments designed to simulate infant mouthing of toys and children’s products. Babich et al. [49] determined migration rates for a range of plasticizers in toys, including PVC articles, and observed rates of up to 4.44 µg/10 cm/min for acetyltributyl citrate (ATBC), corresponding to a median exposure estimate of 1.1 μg/kg/day corrected for infant body mass and mouthing behavior. Polybrominated diphenyl ethers (PBDEs) have also been evidenced to transfer from toys with almost 3% of BDE-209 leached from the surface of a soft plastic figurine following 60 min of simulated mouthing [50]. Ionas et al. [50] reported greater migration of lighter PBDE congeners, while leaching rates appeared to be more dependent on polymer type (hard versus soft) than the PBDE concentration. CP migration during mouthing may be analogous to the findings of Ionas et al. [50], as the molecular mass and logK_OW_ ranges of PBDEs overlap with those of CPs and both groups of flame retardants are applied as non-reactive additives.

Dermal uptake of CPs from consumer products may represent another exposure pathway from items such as yoga mats, pool mats and flip-flops, which are likely to be in direct contact with bare skin for extended periods. Exposure via this pathway is extremely difficult to evaluate because both compound migration from materials and dermal absorption rates must be considered. While absorption characteristics for CPs have not yet been determined, Yuan et al. [51] developed a model for CP exposure via hands using the USEPA’s Exposure Factors Handbook [52] and earlier ex vivo studies of halogenated flame retardant (HFR) uptake [53]. Based on CP measurements in hand wipes from 60 participants, the median daily exposure to SCCPs and MCCPs was estimated to be 3.6 and 11 ng/kg body weight via the skin of hands alone. Ex vivo experimental evidence has shown dermal absorption of chlorinated organophosphate flame retardants at rates up to 28% following a direct finite dose of 500 ng/cm^2^ [54], while uptake of brominated flame retardants (BFRs) has also been indicated [55]. BFR uptake by ex vivo skin samples via contact with treated sofa fabrics was also investigated to illustrate that exposure can indeed occur via direct migration from materials into the skin [56]. Daily exposure estimates by this method were as high as 8.8 and 111 ng/kg bw for ∑penta-BDEs and hexabromocyclododecanes, respectively, for male adults. Evidence of dermal uptake of halogenated compounds with similar properties to CPs suggests that this exposure pathway should be considered in assessments of overall exposure to SCCPs and MCPs, and that the complex mechanisms involved warrant further investigation in the future.

## 4. Conclusions

This study assessed SCCPs and replacement MCCPs in a range of consumer products and toys purchased in 2019 on the Belgian market to determine overall concentrations and potential sources to the materials. These results suggested that SCCPs had only been incorporated intentionally within approximately 4% of analyzed samples, demonstrating a marked decrease in SCCP application compared with products purchased in the EU between 2013 and 2017. The identification of one product containing SCCPs almost 100 times the regulation limit, however, illustrates the difficulty in preventing the import of banned goods from other countries and highlights the need for simple SCCP screening methods. Analysis of congener group profiles implied that frequent detection of low-level SCCP and MCCP quantities in samples may derive from the recycling of CP-treated materials or contamination of goods during manufacturing. Such inadvertent incorporation of CPs into consumer products is likely to continue while regulations contain exemptions for SCCP usage in manufacturing equipment and treated materials continue to enter recycling waste streams. This study also provides evidence that APs have been applied within consumer products and toys on the Belgian market to indicate that these compounds warrant further research attention in the future.

## Figures and Tables

**Table 1:** Consumer product and toy sample details.

Code	Product	Polymer	Country of Manufacture
YM-1	Yoga mat	PVC	China
YM-2	Yoga mat	PVC	n.a.
BB-1	Beach ball	PVC	China
BB-2	Beach ball	PVC	China
PM-1	Inflatable pool mat	PVC	Hong Kong
PM-2	Inflatable pool mat	PVC	China
PM-3	Inflatable pool mat	PVC	China
PM-4	Inflatable pool mat	PVC	United Kingdom
CH	Can holder	PVC	China
JR-1	Jump rope	PVC	Taiwan
JR-2	Jump rope	PVC	China
JR-3	Jump rope	PVC	China
JR-4	Jump rope	PVC	China
EC-1	Electrical cable	PVC	China
EC-2	Electrical cable	PVC	China
FF-1	Flip flop	PVC	China
FF-2	Flip flop	PVC	China
FF-3	Flip flop	PVC	China
FF-4	Flip flop	PVC	China
FF-5	Flip flop	Rubber	Bangladesh
RD-1	Rubber duck	Rubber	China
RD-2	Rubber duck	Rubber	China
RD-3	Rubber duck	Rubber	n.a.
CC-1	Corner cover	Rubber	Denmark
CC-2	Corner cover	Rubber	The Netherlands
CL	Clothesline	n.a.	China
ASM-1	Anti-slip mat	n.a.	The Netherlands
ASM-2	Anti-slip mat	n.a.	China

n.a.—not available.

**Table 2:** Summary concentrations of short-chain chlorinated paraffins (SCCPs) and medium-chain chlorinated paraffins (MCCPs) in consumer product and toy samples (µg/g).

Sample	ΣC10	ΣC11	ΣC12	ΣC13	ΣSCCP	Cl%	ΣC14	ΣC15	ΣC16	ΣC17	ΣMCCP	Cl%
YM-1	5.6	1.0	32	<LOQ	39	64.6	<LOQ	<LOQ	<LOQ	<LOQ	<LOQ	-
YM-2	5.0	<LOQ	39	<LOQ	44	65.3	<LOQ	<LOQ	<LOQ	<LOQ	<LOQ	-
BB-1	2.5	<LOQ	1.0	<LOQ	3.5	61.7	<LOQ	<LOQ	<LOQ	<LOQ	<LOQ	-
BB-2	1.9	<LOQ	<LOQ	<LOQ	1.9	61.0	<LOQ	<LOQ	<LOQ	<LOQ	<LOQ	-
PM-1	2.2	<LOQ	<LOQ	<LOQ	2.2	60.9	7.0	5.1	2.2	<LOQ	14	51.1
PM-2	1.3	<LOQ	<LOQ	<LOQ	1.3	60.9	<LOQ	<LOQ	<LOQ	<LOQ	<LOQ	-
PM-3	7.8	2.2	<LOQ	<LOQ	10	57.6	<LOQ	<LOQ	<LOQ	<LOQ	<LOQ	-
PM-4	2.0	<LOQ	<LOQ	<LOQ	2.0	60.9	<LOQ	<LOQ	<LOQ	<LOQ	<LOQ	-
CH	1.2	<LOQ	<LOQ	<LOQ	1.2	63.1	<LOQ	<LOQ	<LOQ	<LOQ	<LOQ	-
JR-1	1.6	<LOQ	<LOQ	<LOQ	1.6	57.3	<LOQ	<LOQ	<LOQ	<LOQ	<LOQ	-
JR-2	49	22	8.1	2.6	82	53.4	9.0	3.0	2.1	<LOQ	14	50.6
JR-3	2.5	<LOQ	<LOQ	<LOQ	2.5	60.6	<LOQ	<LOQ	<LOQ	<LOQ	<LOQ	-
JR-4	2.5	<LOQ	<LOQ	<LOQ	2.5	61.3	<LOQ	<LOQ	<LOQ	<LOQ	<LOQ	-
EC-1	1.5	<LOQ	<LOQ	<LOQ	1.5	60.9	<LOQ	<LOQ	<LOQ	<LOQ	<LOQ	-
EC-2	1.4	<LOQ	<LOQ	<LOQ	1.4	60.9	<LOQ	<LOQ	<LOQ	<LOQ	<LOQ	-
FF-1	2.5	<LOQ	<LOQ	<LOQ	2.5	60.9	<LOQ	<LOQ	<LOQ	<LOQ	<LOQ	-
FF-2	7.3	1.8	<LOQ	<LOQ	9.1	57.5	<LOQ	<LOQ	<LOQ	<LOQ	<LOQ	-
FF-3	1.7	<LOQ	<LOQ	<LOQ	1.7	60.7	<LOQ	<LOQ	<LOQ	<LOQ	<LOQ	-
FF-4	1.6	<LOQ	<LOQ	<LOQ	1.6	60.9	<LOQ	<LOQ	<LOQ	<LOQ	<LOQ	-
FF-5	4.0	<LOQ	<LOQ	<LOQ	4.0	59.3	<LOQ	<LOQ	<LOQ	<LOQ	<LOQ	-
RD-1	5.6	<LOQ	1.8	<LOQ	7.4	60.0	130	83	79	53	350	51.0
RD-2	4.9	<LOQ	<LOQ	<LOQ	4.9	60.8	<LOQ	<LOQ	<LOQ	<LOQ	<LOQ	-
RD-3	6.7	<LOQ	1.3	<LOQ	8.0	59.7	5.8	<LOQ	<LOQ	<LOQ	5.8	54.4
CC-1	2.0	<LOQ	<LOQ	<LOQ	2.0	55.8	<LOQ	<LOQ	<LOQ	<LOQ	<LOQ	-
CC-2	<10	<LOQ	<LOQ	<LOQ	<LOQ	-	<LOQ	<LOQ	<LOQ	<LOQ	<LOQ	-
CL	36,000	34,000	31,000	29,000	130,000	57.0	1000	990	890	640	3500	56.7
ASM-1	3.2	1.6	52	<LOQ	57	65.3	<LOQ	<LOQ	<LOQ	<LOQ	<LOQ	-
ASM-2	7.5	1.4	31	<LOQ	40	64.4	<LOQ	<LOQ	<LOQ	<LOQ	<LOQ	-
%DF	93	21	32	7.0	96	-	18	14	14	7.0	18	-
Median	2.5	<LOQ	<LOQ	<LOQ	2.5	-	<LOQ	<LOQ	<LOQ	<LOQ	<LOQ	-
Min	<LOQ	<LOQ	<LOQ	<LOQ	<LOQ	-	<LOQ	<LOQ	<LOQ	<LOQ	<LOQ	-
Max	36,000	34,000	31,000	29,000	130,000	-	1000	990	890	640	3500	-

## Data Availability

All of the data is presented in the article and associated Supplementary Materials.

## Supplementary Materials

The following are available online at https://www.mdpi.com/1660-4601/18/3/1069/s1, Section S1: Images of consumer goods samples purchased on the Belgian market. Section S2: Determination of congener percentage compositions in medium-chain chlorinated paraffin (MCCP) technical mixtures with 42 and 57% chlorine contents. Table S1: LC-ESI/MS/MS instrumental acquisition parameters. Table S2: GC–EI-Orbitrap-HRMS extracted ions used for the measurement of MCCP congeners. Table S3: Congener percentage compositions in technical mixtures of SCCP 55.5 and 63%Cl. Table S4: Congener percentage compositions in technical mixtures of MCCP 42 and 57%Cl. Table S5: Concentrations (μg/g) of SCCPs and MCCPs in four samples of polyvinyl chloride (PVC) containing SCCPs and MCCPs by ultrasonication in both toluene and tetrahydrofuran (THF). Table S6: Concentrations of short-chain chlorinated paraffins (SCCPs) in polymer samples (μg/g). Table S7: Concentrations of medium-chain chlorinated paraffins (MCCPs) in polymer samples (μg/g). Table S8: Plasticizers detected in consumer products and toys by GC-EI/MS.
