# Supplementary material for: Short- and Medium-Chain Chlorinated Paraffins in Polyvinylchloride and Rubber Consumer Products and Toys Purchased on the Belgian Market
Source: Int J Environ Res Public Health. 2021 Jan 26;18(3):1069. doi: 10.3390/ijerph18031069 (PMC7908593; doi:10.3390/ijerph18031069)
Supplement: Supplementary file 1 [file ijerph-18-01069-s001.pdf]

Supplementary Information

Short- and medium-chain chlorinated paraffins in polyvinyl chloride and rubber consumer products and toys purchased on the Belgian market

Thomas J. McGrath[a,*], Giulia Poma[a], Hidenori Matsukami[b], Govindan Malarvannan[a], Natsuko Kajiwara[b], Adrian Covaci[a,**]

[a]Toxicological Centre, University of Antwerp, Universiteitsplein 1, 2610, Wilrijk, Belgium

[b]Center for Material Cycles and Waste Management Research, National Institute for Environmental Studies (NIES), 16-2 Onogawa, Tsukuba, 305-8506, Japan

corresponding authors:
*thomas.jacob.mcgrath@gmail.com
**adrian.covaci@uantwerpen.be

Section S1. Images of consumer goods samples purchased on the Belgian market.

YM-1 (yoga mat)

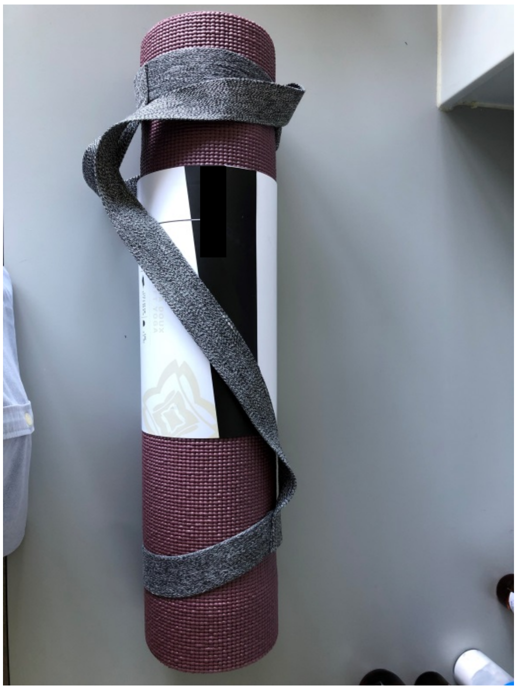

YM-2 (yoga mat)

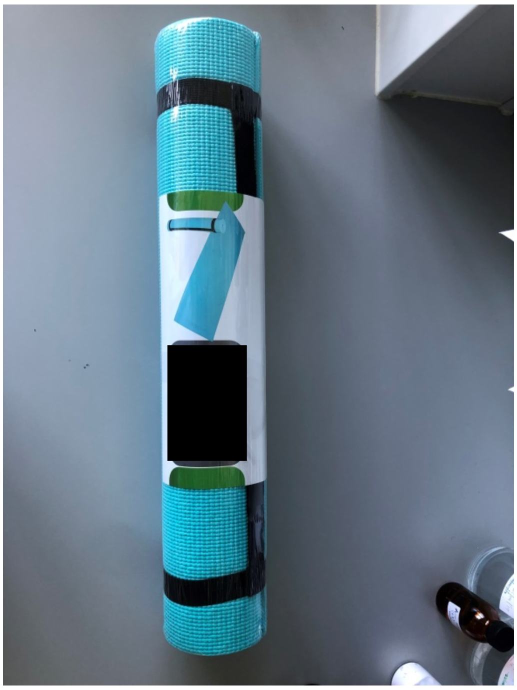

BB-1 (beach ball)

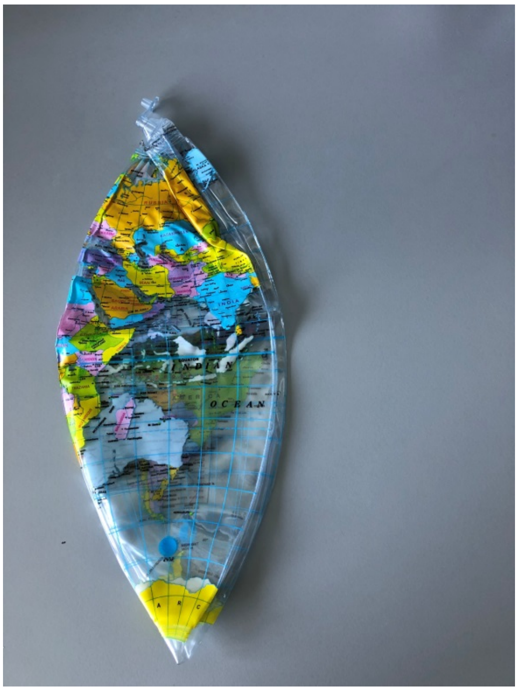

BB-2 (beach ball)

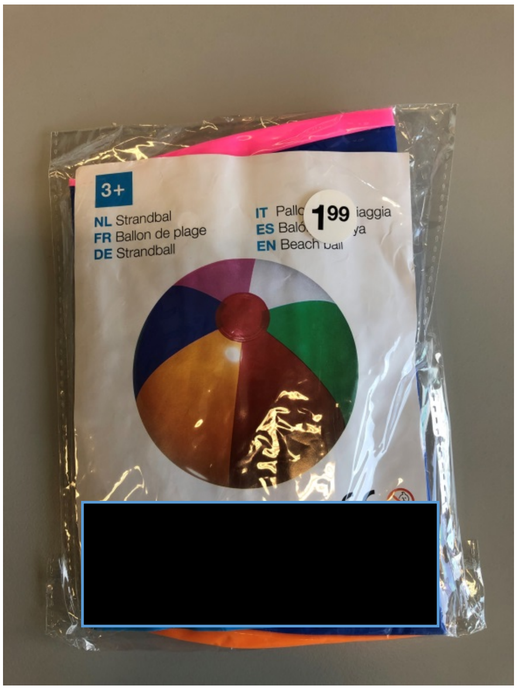

PM-1 (pool mat)

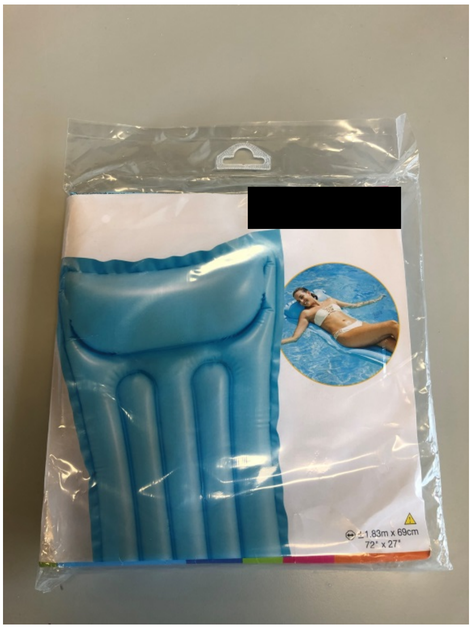

PM-2 (pool mat)

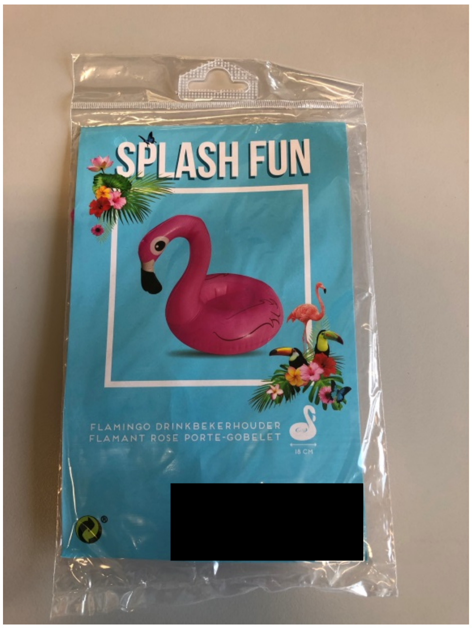

PM-3 (pool mat)

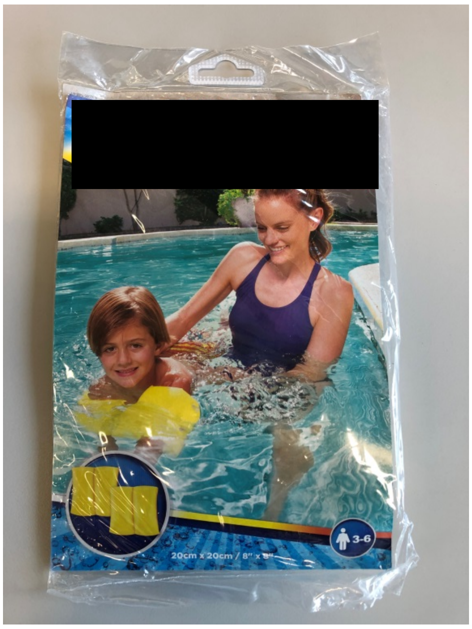

PM-4 (pool mat)

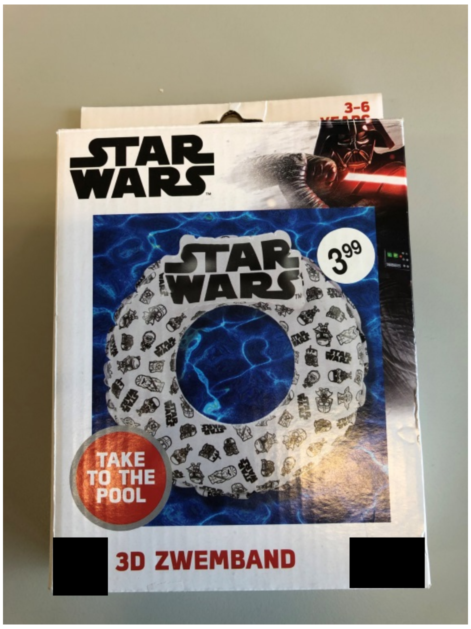

CH (cup holder)

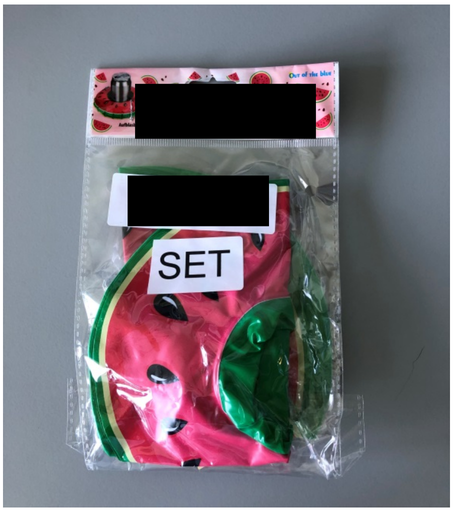

JR-1 (jump rope)

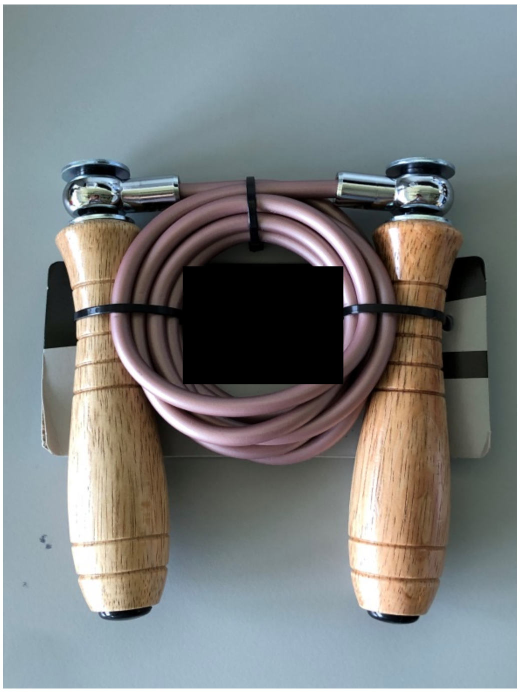

JR-2 (jump rope)

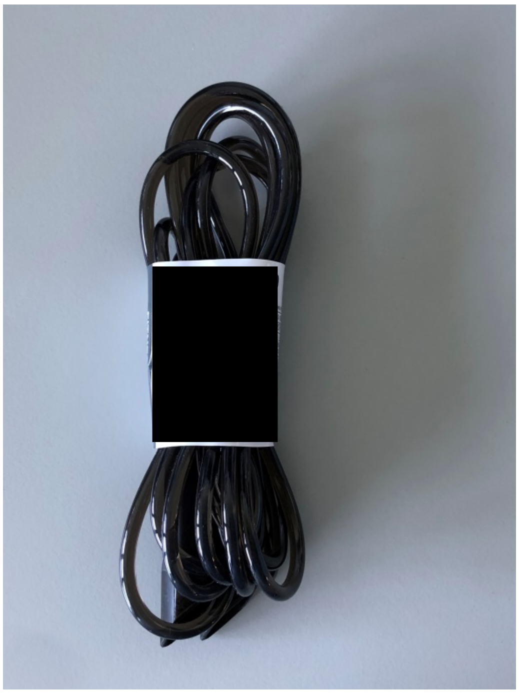

JR-3 (jump rope)

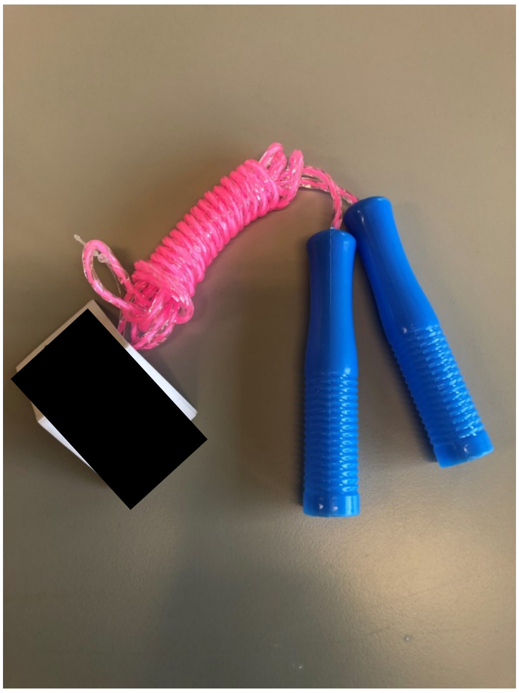

JR-4 (jump rope)

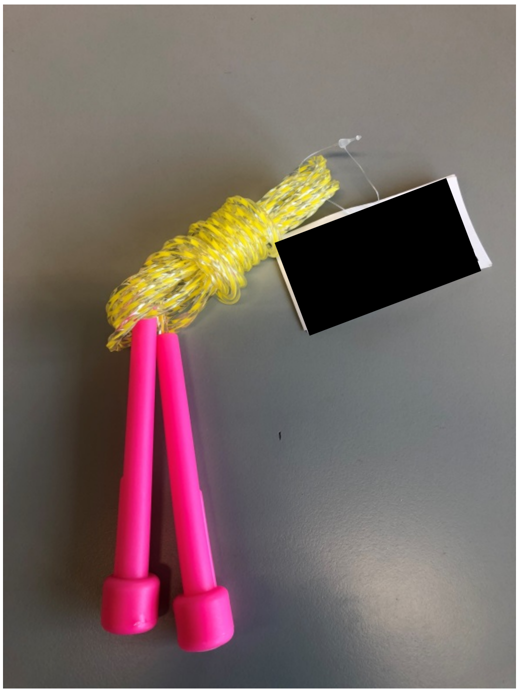

EC-1 (electrical cable)

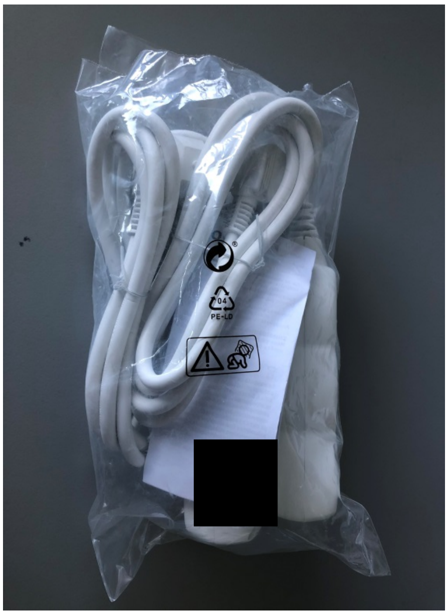

EC-2 (electrical cable)

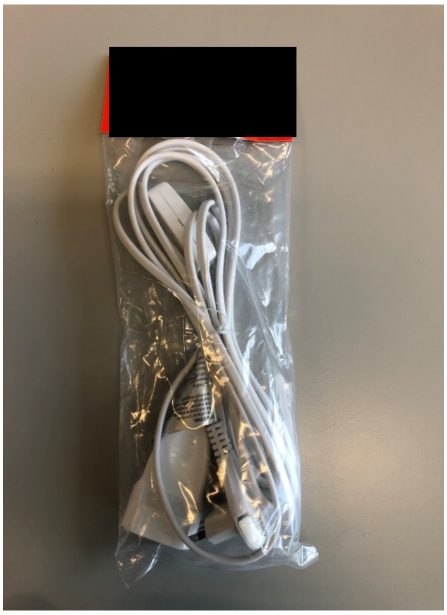

FF-1 (flip flop)

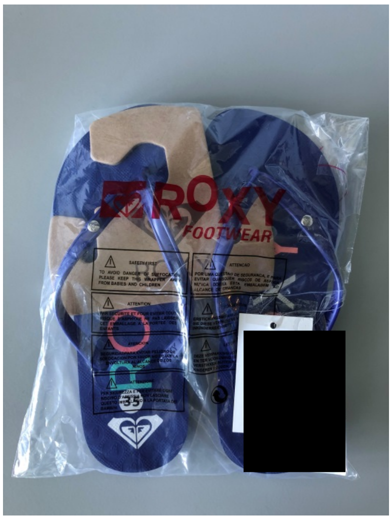

FF-2 (flip flop)

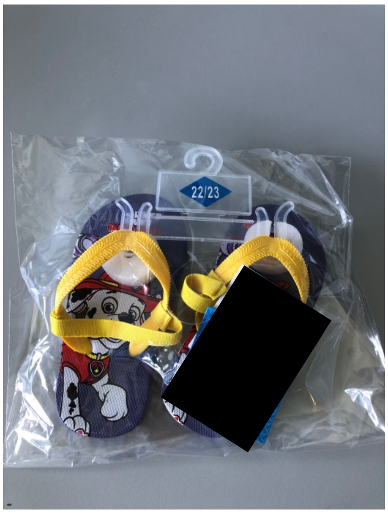

FF-3 (flip flop)

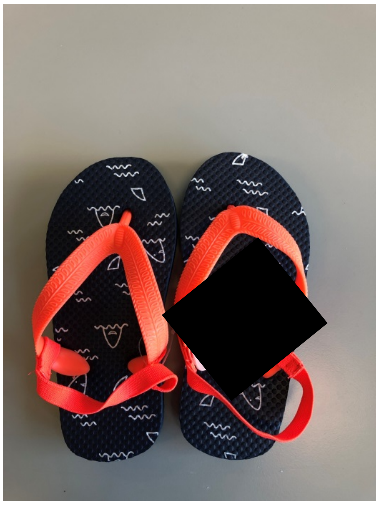

FF-4 (flip flop)

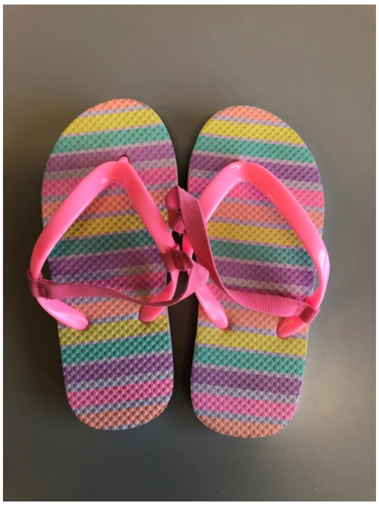

FF-5 (flip flop)

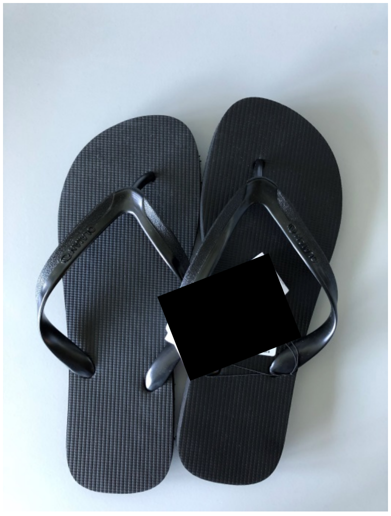

RD-1 (rubber duck)

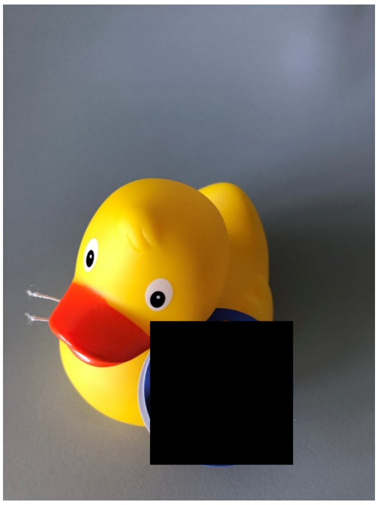

RD-2 (rubber duck)

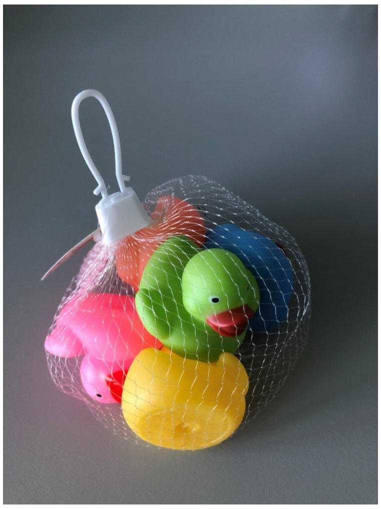

RD-3 (rubber duck)

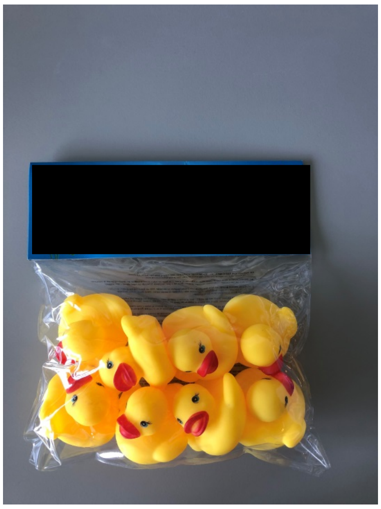

CC-1 (corner cover)

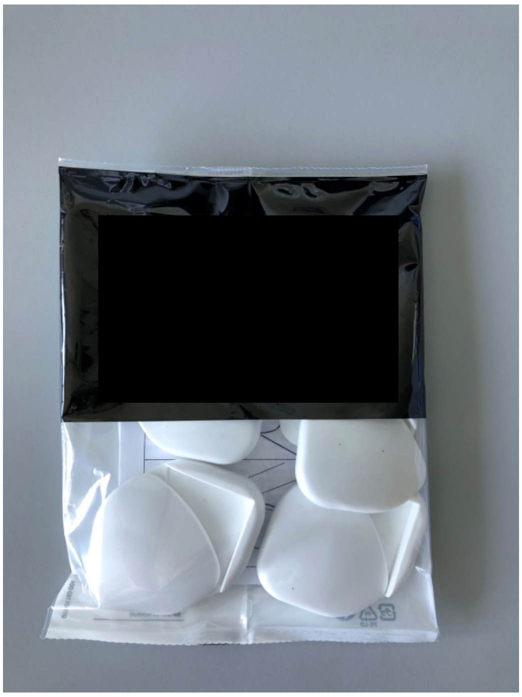

CC-2 (corner cover)

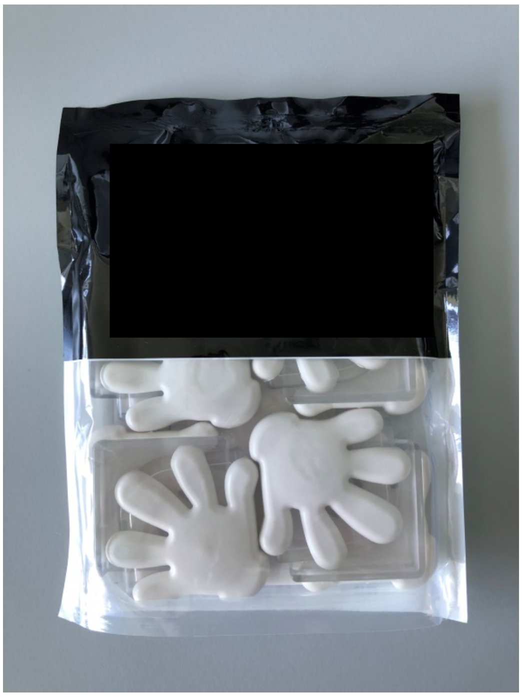

CL (clothesline)

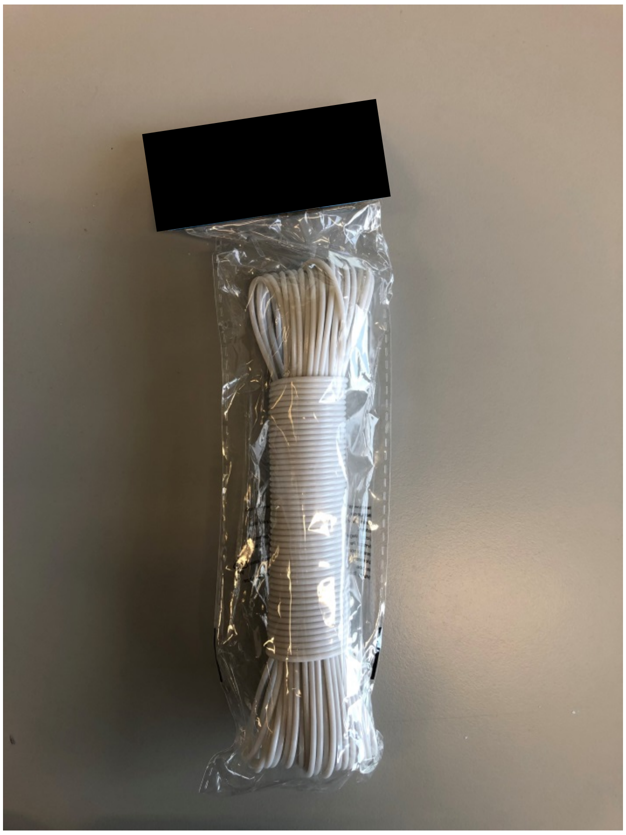

ASM-1 (anti-slip mat)

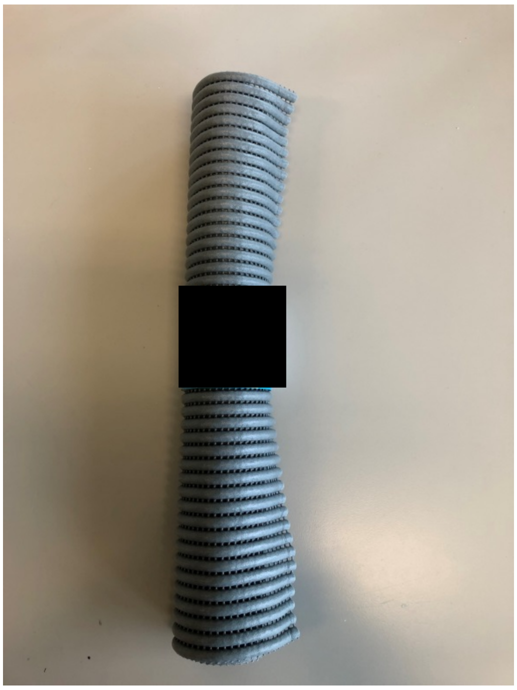

ASM-2 (anti-slip mat)

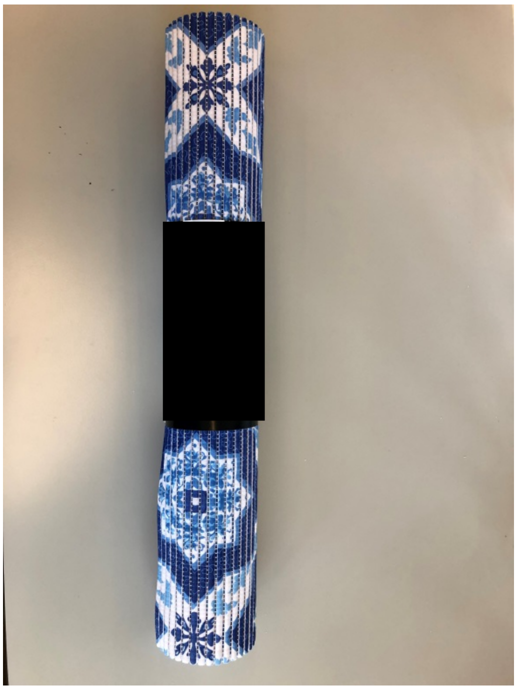

Table S1. LC-ESI/MS/MS instrumental acquisition parameters.

	Congener group	Chemical formula	RT (min)	MRM 1		MRM 2	
				m/z	CE (eV)	*m/z*	CE (eV)
C10	CP 10-4	$C_{10}H_{18}Cl_4$	2.88	339 > 59	10	337 > 59	10
	CP 10-5	$C_{10}H_{17}Cl_5$	3.12	373 > 59	20	371 > 59	20
	CP 10-6	$C_{10}H_{16}Cl_6$	3.45	407 > 347	5	405 > 345	5
	CP 10-7	$C_{10}H_{15}Cl_7$	3.83	441 > 381	5	439 > 379	5
	CP 10-8	$C_{10}H_{14}Cl_8$	4.32	477 > 417	5	475 > 415	5
C11	CP 11-4	$C_{11}H_{20}Cl_4$	3.32	353 > 59	10	351 > 59	10
	CP 11-5	$C_{11}H_{19}Cl_5$	3.61	387 > 59	25	385 > 59	25
	CP 11-6	$C_{11}H_{18}Cl_6$	3.98	421 > 361	5	419 > 359	5
	CP 11-7	$C_{11}H_{17}Cl_7$	4.39	455 > 395	5	453 > 393	5
	CP 11-8	$C_{11}H_{16}Cl_8$	4.83	491 > 431	5	489 > 429	5
	CP 11-9	$C_{11}H_{15}Cl_9$	5.32	525 > 465	5	523 > 463	5
C12	CP 12-4	$C_{12}H_{22}Cl_4$	3.87	367 > 59	10	363 > 59	10
	CP 12-5	$C_{12}H_{21}Cl_5$	4.15	401 > 59	25	399 > 59	25
	CP 12-6	$C_{12}H_{20}Cl_6$	4.53	435 > 375	5	433 > 373	5
	CP 12-7	$C_{12}H_{19}Cl_7$	4.90	469 > 409	5	467 > 407	5
	CP 12-8	$C_{12}H_{18}Cl_8$	5.35	505 > 445	5	503 > 443	5
	CP 12-9	$C_{12}H_{17}Cl_9$	5.79	539 > 479	5	537 > 477	5
	CP 12-10	$C_{12}H_{16}Cl_{10}$	6.22	573 > 513	5	571 > 511	5
C13	CP 13-4	$C_{13}H_{24}Cl_4$	4.45	381 > 59	15	379 > 59	15
	CP 13-5	$C_{13}H_{23}Cl_5$	4.73	415 > 59	25	413 > 59	25
	CP 13-6	$C_{13}H_{22}Cl_6$	5.10	449 > 389	5	447 > 387	5
	CP 13-7	$C_{13}H_{21}Cl_7$	5.46	483 > 423	5	481 > 421	5
	CP 13-8	$C_{13}H_{20}Cl_8$	5.87	519 > 459	5	517 > 457	5
	CP 13-9	$C_{13}H_{19}Cl_9$	6.31	553 > 493	5	551 > 491	5
	CP 13-10	$C_{13}H_{18}Cl_{10}$	6.72	587 > 527	5	585 > 525	5
C14	CP 14-4	$C_{14}H_{26}Cl_4$	5.04	395 > 59	10	393 > 59	10
	CP 14-5	$C_{14}H_{25}Cl_5$	5.27	429 > 59	15	427 > 59	15
	CP 14-6	$C_{14}H_{24}Cl_6$	5.58	463 > 59	20	461 > 59	20
	CP 14-7	$C_{14}H_{23}Cl_7$	5.95	497 > 437	5	495 > 435	5
	CP 14-8	$C_{14}H_{22}Cl_8$	6.32	533 > 473	5	531 > 471	5
	CP 14-9	$C_{14}H_{21}Cl_9$	6.73	567 > 507	5	565 > 505	5
	CP 14-10	$C_{14}H_{20}Cl_{10}$	7.15	601 > 541	5	599 > 539	5
C15	CP 15-4	$C_{15}H_{28}Cl_4$	5.59	409 > 59	10	407 > 59	10
	CP 15-5	$C_{15}H_{27}Cl_5$	5.84	443 > 59	20	441 > 59	20
	CP 15-6	$C_{15}H_{26}Cl_6$	6.12	477 > 59	20	475 > 59	20
	CP 15-7	$C_{15}H_{25}Cl_7$	6.48	511 > 451	5	509 > 449	5
	CP 15-8	$C_{15}H_{24}Cl_8$	6.81	547 > 487	5	545 > 485	5
	CP 15-9	$C_{15}H_{23}Cl_9$	7.19	581 > 521	5	579 > 519	5
	CP 15-10	$C_{15}H_{22}Cl_{10}$	7.57	615 > 555	5	613 > 553	5
C16	CP 16-4	$C_{16}H_{30}Cl_4$	6.22	423 > 59	10	421 > 59	10
	CP 16-5	$C_{16}H_{29}Cl_5$	6.39	457 > 59	20	455 > 59	20
	CP 16-6	$C_{16}H_{28}Cl_6$	6.63	491 > 59	20	489 > 59	20
	CP 16-7	$C_{16}H_{27}Cl_7$	6.93	525 > 465	5	523 > 463	5
	CP 16-8	$C_{16}H_{26}Cl_8$	7.26	561 > 501	5	559 > 499	5
	CP 16-9	$C_{16}H_{25}Cl_9$	7.60	595 > 535	5	593 > 533	5
	CP 16-10	$C_{16}H_{24}Cl_{10}$	7.97	629 > 569	5	627 > 567	5
C17	CP 17-4	$C_{17}H_{32}Cl_4$	6.79	437 > 59	10	435 > 59	10
	CP 17-5	$C_{17}H_{31}Cl_5$	6.92	471 > 59	20	469 > 59	20
	CP 17-6	$C_{17}H_{30}Cl_6$	7.13	505 > 59	20	503 > 59	20
	CP 17-7	$C_{17}H_{29}Cl_7$	7.41	539 > 479	5	537 > 477	5
	CP 17-8	$C_{17}H_{28}Cl_8$	7.70	575 > 515	5	573 > 513	5
	CP 17-9	$C_{17}H_{27}Cl_9$	8.02	609 > 549	5	607 > 547	5
	CP 17-10	$C_{17}H_{26}Cl_{10}$	8.36	643 > 583	5	641 > 581	5

MRM = multiple reaction monitoring, RT = retention time, CE = collision energy

Section S2. Determination of congener percentage compositions in medium-chain chlorinated paraffin (MCCP) technical mixtures with 42 and 57% chlorine contents.

Congener percentage compositions in the technical mixtures of MCCPs 42 and 57 %Cl were determined by following the method of Matsukami et al. (2020) developed for those of SCCPs. A gas chromatography (GC)-electron ionization (EI)-Orbitrap-high-resolution mass spectrometry (HRMS) system (Q Exactive GC; Thermo Fisher Scientific, Inc., Waltham, MA, USA) equipped with a DB-5MS column (15 m × 0.25 mm, i.d. 0.1 mm; Agilent Technologies Inc., Santa Clara, CA, USA) was used to measure the MCCP congeners. The GC oven temperature program was as follows: initial 100 °C, hold for 5 min, ramp to 300 °C at 20 °C/min, hold for 10 min; ionization voltage, 70 eV; source temperature, 300 °C; mass range, m/z 150–700. The interface temperature was set to 300 °C. The resolving power was set at 60,000 full width at half maximum. Helium was used as the carrier gas. The flow rate of the carrier gas was set to 1 mL/min. The GC injection volume was 2 μL with an injector temperature of 280 °C. Table S2 presents the ions extracted with m/z widths of ±5 ppm for the measurement of MCCP congeners.

Table S2. GC–EI-Orbitrap-HRMS extracted ions used for the measurement of MCCP congeners.

Congener	Type of ion	M	M+2	M+4
$C_{14}Cl_3$	$[M-Cl-2HCl]^+$	193.19508		
$C_{14}Cl_4$	$[M-Cl-2HCl]^+$	227.15611	229.15316	
$C_{14}Cl_5$	$[M-Cl-2HCl]^+$	261.11713	263.11419	
$C_{14}Cl_6$	$[M-Cl-2HCl]^+$	295.07816	297.07522	
$C_{14}Cl_7$	$[M-Cl-2HCl]^+$	329.03919	331.03632	
$C_{14}Cl_8$	$[M-Cl-2HCl]^+$		364.99733	366.99448
$C_{14}Cl_9$	$[M-Cl-2HCl]^+$		398.95835	400.95548
$C_{14}Cl_{10}$	$[M-Cl-2HCl]^+$		432.91937	434.91648
$C_{14}Cl_{11}$	$[M-Cl-2HCl]^+$		466.88039	468.87749
$C_{15}Cl_3$	$[M-Cl-2HCl]^+$	207.21073		
$C_{15}Cl_4$	$[M-Cl-2HCl]^+$	241.17176	243.16881	
$C_{15}Cl_5$	$[M-Cl-2HCl]^+$	275.13278	277.12984	
$C_{15}Cl_6$	$[M-Cl-2HCl]^+$	309.09381	311.09088	
$C_{15}Cl_7$	$[M-Cl-2HCl]^+$	343.05484	345.05198	
$C_{15}Cl_8$	$[M-Cl-2HCl]^+$		379.01299	381.01016
$C_{15}Cl_9$	$[M-Cl-2HCl]^+$		412.97401	414.97115
$C_{15}Cl_{10}$	$[M-Cl-2HCl]^+$		446.93503	448.93215
$C_{15}Cl_{11}$	$[M-Cl-2HCl]^+$		480.89605	482.89316
$C_{16}Cl_3$	$[M-Cl-2HCl]^+$	221.22638		
$C_{16}Cl_4$	$[M-Cl-2HCl]^+$	255.18741	257.18446	
$C_{16}Cl_5$	$[M-Cl-2HCl]^+$	289.14843	291.14549	
$C_{16}Cl_6$	$[M-Cl-2HCl]^+$	323.10946	325.10653	
$C_{16}Cl_7$	$[M-Cl-2HCl]^+$	357.07049	359.06765	
$C_{16}Cl_8$	$[M-Cl-2HCl]^+$		393.02865	395.02583
$C_{16}Cl_9$	$[M-Cl-2HCl]^+$		426.98967	428.98682
$C_{16}Cl_{10}$	$[M-Cl-2HCl]^+$		460.95068	462.94782
$C_{16}Cl_{11}$	$[M-Cl-2HCl]^+$		494.91170	496.90882
$C_{17}Cl_3$	$[M-Cl-2HCl]^+$	235.24203		
$C_{17}Cl_4$	$[M-Cl-2HCl]^+$	269.20306	271.20011	
$C_{17}Cl_5$	$[M-Cl-2HCl]^+$	303.16408	305.16115	
$C_{17}Cl_6$	$[M-Cl-2HCl]^+$	337.12511	339.12219	
$C_{17}Cl_7$	$[M-Cl-2HCl]^+$	371.08614	373.08331	
$C_{17}Cl_8$	$[M-Cl-2HCl]^+$		407.04432	409.04151
$C_{17}Cl_9$	$[M-Cl-2HCl]^+$		441.00533	443.00249
$C_{17}Cl_{10}$	$[M-Cl-2HCl]^+$		474.96634	476.96349
$C_{17}Cl_{11}$	$[M-Cl-2HCl]^+$		508.92736	510.92449

Table S3. Congener percentage compositions in technical mixtures of SCCP 55.5 and 63 %Cl.

Congener	SCCP 55.5% Cl	SCCP 63% Cl	1:1 mixture of SCCP 55.5% and 63% Cl
$C_{10}Cl_3$	0	0	0
$C_{10}Cl_4$	1.81	0.06	0.93
$C_{10}Cl_5$	3.66	0.82	2.24
$C_{10}Cl_6$	1.93	3.08	2.51
$C_{10}Cl_7$	0.49	2.93	1.71
$C_{10}Cl_8$	0.07	1.05	0.56
$C_{10}Cl_9$	0	0.17	0.09
$C_{10}Cl_{10}$	0	0	0
$C_{11}Cl_3$	0	0	0
$C_{11}Cl_4$	5.23	0.05	2.64
$C_{11}Cl_5$	15.51	1.28	8.39
$C_{11}Cl_6$	12.29	6.72	9.50
$C_{11}Cl_7$	4.06	12.77	8.41
$C_{11}Cl_8$	0.95	9.86	5.40
$C_{11}Cl_9$	0.11	2.95	1.53
$C_{11}Cl_{10}$	0.01	0.27	0.14
$C_{11}Cl_{11}$	0	0	0
$C_{12}Cl_3$	0	0	0
$C_{12}Cl_4$	2.74	0.00	1.37
$C_{12}Cl_5$	11.07	0.28	5.68
$C_{12}Cl_6$	12.51	2.82	7.67
$C_{12}Cl_7$	5.82	8.31	7.06
$C_{12}Cl_8$	1.83	12.30	7.06
$C_{12}Cl_9$	0.33	7.14	3.73
$C_{12}Cl_{10}$	0.03	1.38	0.70
$C_{12}Cl_{11}$	0	0.09	0.04
$C_{13}Cl_3$	0	0	0
$C_{13}Cl_4$	0.79	0	0.39
$C_{13}Cl_5$	4.02	0	2.01
$C_{13}Cl_6$	6.28	0.46	3.37
$C_{13}Cl_7$	5.44	4.05	4.75
$C_{13}Cl_8$	2.43	9.64	6.03
$C_{13}Cl_9$	0.55	8.24	4.39
$C_{13}Cl_{10}$	0.07	2.97	1.52
$C_{13}Cl_{11}$	0	0.31	0.16
SCCPs	100	100	100
Cl (%)	56.1	62.7	59.9

Table S4. Congener percentage compositions in technical mixtures of MCCP 42 and 57 %Cl.

Congener	MCCP 42% Cl	MCCP 57% Cl	1:1 mixture of MCCP 42% and 57% Cl
$C_{14}Cl_3$	4.09	0	2.05
$C_{14}Cl_4$	19.15	0.09	9.62
$C_{14}Cl_5$	28.41	2.11	15.26
$C_{14}Cl_6$	15.72	11.43	13.58
$C_{14}Cl_7$	3.74	22.75	13.11
$C_{14}Cl_8$	0.43	20.00	10.22
$C_{14}Cl_9$	0.02	7.80	3.91
$C_{14}Cl_{10}$	0	1.34	0.67
$C_{14}Cl_{11}$	0	0.09	0.05
$C_{15}Cl_3$	0.89	0	0.45
$C_{15}Cl_4$	4.91	0.01	2.46
$C_{15}Cl_5$	9.53	0.34	4.94
$C_{15}Cl_6$	6.59	2.45	4.52
$C_{15}Cl_7$	2.04	7.30	4.67
$C_{15}Cl_8$	0.26	9.03	4.65
$C_{15}Cl_9$	0.02	4.99	2.51
$C_{15}Cl_{10}$	0	1.21	0.61
$C_{15}Cl_{11}$	0	0.11	0.06
$C_{16}Cl_3$	0.09	0	0.05
$C_{16}Cl_4$	0.57	0	0.29
$C_{16}Cl_5$	1.37	0	0.69
$C_{16}Cl_6$	1.16	0.34	0.75
$C_{16}Cl_7$	0.42	1.47	0.95
$C_{16}Cl_8$	0.06	2.51	1.29
$C_{16}Cl_9$	0	1.88	0.94
$C_{16}Cl_{10}$	0	0.60	0.30
$C_{16}Cl_{11}$	0	0.07	0.04
$C_{17}Cl_3$	0	0	0
$C_{17}Cl_4$	0.06	0	0.03
$C_{17}Cl_5$	0.19	0	0.10
$C_{17}Cl_6$	0.19	0	0.10
$C_{17}Cl_7$	0.08	0.32	0.20
$C_{17}Cl_8$	0.01	0.74	0.38
$C_{17}Cl_9$	0	0.70	0.35
$C_{17}Cl_{10}$	0	0.26	0.13
$C_{17}Cl_{11}$	0	0.04	0.02
MCCPs	100	100	100
Cl (%)	46.8	57.2	52.5

Table S5. Concentrations (μg/g) of SCCPs and MCCPs in four samples of polyvinyl chloride (PVC) containing SCCPs and MCCPs by ultrasonication in both toluene and tetrahydrofuran (THF).

Sample	SCCPs		MCCPs	
	Toluene	THF	Toluene	THF
PVC-A	190	220	1,700	1,700
PVC-B	510	630	44,000	43,000
PVC-C	6,400	7,100	4,200	4,200
PVC-D	5,800	7,700	43,000	49,000

Table S6. Concentrations of short-chain chlorinated paraffins (SCCPs) in polymer samples (μg/g).

Congener group		LOQ	YM-1	YM-2	BB-1	BB-2	PM-1	PM-2	PM-3	PM-4	CH	JR-1	JR-2	JR-3	JR-4	EC-1	EC-2	FF-1	FF-2	FF-3	FF-4	FF-5
C10	Cl4	10	<10	<10	<10	<10	<10	<10	<10	<10	<10	<10	20	<10	<10	<10	<10	<10	<10	<10	<10	<10
	Cl5	1.0	<1.0	<1.0	<1.0	<1.0	<1.0	<1.0	4.1	<1.0	<1.0	<1.0	19	<1.0	<1.0	<1.0	<1.0	<1.0	3.9	<1.0	<1.0	1.2
	Cl6	1.0	5.6	5.0	2.5	1.9	2.2	1.3	3.7	2.0	1.2	1.6	8.6	2.5	2.5	1.5	1.4	2.5	3.4	1.7	1.6	2.8
	Cl7	1.0	<1.0	<1.0	<1.0	<1.0	<1.0	<1.0	<1.0	<1.0	<1.0	<1.0	1.1	<1.0	<1.0	<1.0	<1.0	<1.0	<1.0	<1.0	<1.0	<1.0
	Cl8	1.0	<1.0	<1.0	<1.0	<1.0	<1.0	<1.0	<1.0	<1.0	<1.0	<1.0	<1.0	<1.0	<1.0	<1.0	<1.0	<1.0	<1.0	<1.0	<1.0	<1.0
C11	Cl4	5.0	<5.0	<5.0	<5.0	<5.0	<5.0	<5.0	<5.0	<5.0	<5.0	<5.0	9.3	<5.0	<5.0	<5.0	<5.0	<5.0	<5.0	<5.0	<5.0	<5.0
	Cl5	1.0	<1.0	<1.0	<1.0	<1.0	<1.0	<1.0	2.2	<1.0	<1.0	<1.0	9.6	<1.0	<1.0	<1.0	<1.0	<1.0	1.8	<1.0	<1.0	<1.0
	Cl6	1.0	<1.0	<1.0	<1.0	<1.0	<1.0	<1.0	<1.0	<1.0	<1.0	<1.0	3.0	<1.0	<1.0	<1.0	<1.0	<1.0	<1.0	<1.0	<1.0	<1.0
	Cl7	1.0	<1.0	<1.0	<1.0	<1.0	<1.0	<1.0	<1.0	<1.0	<1.0	<1.0	<1.0	<1.0	<1.0	<1.0	<1.0	<1.0	<1.0	<1.0	<1.0	<1.0
	Cl8	1.0	<1.0	<1.0	<1.0	<1.0	<1.0	<1.0	<1.0	<1.0	<1.0	<1.0	<1.0	<1.0	<1.0	<1.0	<1.0	<1.0	<1.0	<1.0	<1.0	<1.0
	Cl9	1.0	1.0	<1.0	<1.0	<1.0	<1.0	<1.0	<1.0	<1.0	<1.0	<1.0	<1.0	<1.0	<1.0	<1.0	<1.0	<1.0	<1.0	<1.0	<1.0	<1.0
C12	Cl4	2.0	<2.0	<2.0	<2.0	<2.0	<2.0	<2.0	<2.0	<2.0	<2.0	<2.0	4.6	<2.0	<2.0	<2.0	<2.0	<2.0	<2.0	<2.0	<2.0	<2.0
	Cl5	1.0	<1.0	<1.0	<1.0	<1.0	<1.0	<1.0	<1.0	<1.0	<1.0	<1.0	3.5	<1.0	<1.0	<1.0	<1.0	<1.0	<1.0	<1.0	<1.0	<1.0
	Cl6	1.0	<1.0	<1.0	<1.0	<1.0	<1.0	<1.0	<1.0	<1.0	<1.0	<1.0	<1.0	<1.0	<1.0	<1.0	<1.0	<1.0	<1.0	<1.0	<1.0	<1.0
	Cl7	1.0	1.8	<1.0	1.0	<1.0	<1.0	<1.0	<1.0	<1.0	<1.0	<1.0	<1.0	<1.0	<1.0	<1.0	<1.0	<1.0	<1.0	<1.0	<1.0	<1.0
	Cl8	1.0	12	12	<1.0	<1.0	<1.0	<1.0	<1.0	<1.0	<1.0	<1.0	<1.0	<1.0	<1.0	<1.0	<1.0	<1.0	<1.0	<1.0	<1.0	<1.0
	Cl9	1.0	15	20	<1.0	<1.0	<1.0	<1.0	<1.0	<1.0	<1.0	<1.0	<1.0	<1.0	<1.0	<1.0	<1.0	<1.0	<1.0	<1.0	<1.0	<1.0
	Cl10	1.0	2.9	7.3	<1.0	<1.0	<1.0	<1.0	<1.0	<1.0	<1.0	<1.0	<1.0	<1.0	<1.0	<1.0	<1.0	<1.0	<1.0	<1.0	<1.0	<1.0
C13	Cl4	1.0	<1.0	<1.0	<1.0	<1.0	<1.0	<1.0	<1.0	<1.0	<1.0	<1.0	1.6	<1.0	<1.0	<1.0	<1.0	<1.0	<1.0	<1.0	<1.0	<1.0
	Cl5	1.0	<1.0	<1.0	<1.0	<1.0	<1.0	<1.0	<1.0	<1.0	<1.0	<1.0	1.0	<1.0	<1.0	<1.0	<1.0	<1.0	<1.0	<1.0	<1.0	<1.0
	Cl6	1.0	<1.0	<1.0	<1.0	<1.0	<1.0	<1.0	<1.0	<1.0	<1.0	<1.0	<1.0	<1.0	<1.0	<1.0	<1.0	<1.0	<1.0	<1.0	<1.0	<1.0
	Cl7	1.0	<1.0	<1.0	<1.0	<1.0	<1.0	<1.0	<1.0	<1.0	<1.0	<1.0	<1.0	<1.0	<1.0	<1.0	<1.0	<1.0	<1.0	<1.0	<1.0	<1.0
	Cl8	1.0	<1.0	<1.0	<1.0	<1.0	<1.0	<1.0	<1.0	<1.0	<1.0	<1.0	<1.0	<1.0	<1.0	<1.0	<1.0	<1.0	<1.0	<1.0	<1.0	<1.0
	Cl9	1.0	<1.0	<1.0	<1.0	<1.0	<1.0	<1.0	<1.0	<1.0	<1.0	<1.0	<1.0	<1.0	<1.0	<1.0	<1.0	<1.0	<1.0	<1.0	<1.0	<1.0
	Cl10	1.0	<1.0	<1.0	<1.0	<1.0	<1.0	<1.0	<1.0	<1.0	<1.0	<1.0	<1.0	<1.0	<1.0	<1.0	<1.0	<1.0	<1.0	<1.0	<1.0	<1.0
Summary	ΣC10		5.6	5.0	2.5	1.9	2.2	1.3	7.8	2.0	1.2	1.6	49	2.5	2.5	1.5	1.4	2.5	7.3	1.7	1.6	4.0
	ΣC11		1.0	<LOQ	<LOQ	<LOQ	<LOQ	<LOQ	2.2	<LOQ	<LOQ	<LOQ	22	<LOQ	<LOQ	<LOQ	<LOQ	<LOQ	1.8	<LOQ	<LOQ	<LOQ
	ΣC12		32	39	1.0	<LOQ	<LOQ	<LOQ	<LOQ	<LOQ	<LOQ	<LOQ	8.1	<LOQ	<LOQ	<LOQ	<LOQ	<LOQ	<LOQ	<LOQ	<LOQ	<LOQ
	ΣC13		<LOQ	<LOQ	<LOQ	<LOQ	<LOQ	<LOQ	<LOQ	<LOQ	<LOQ	<LOQ	2.6	<LOQ	<LOQ	<LOQ	<LOQ	<LOQ	<LOQ	<LOQ	<LOQ	<LOQ
	ΣSCCP		39	44	3.5	1.9	2.2	1.3	10	2.0	1.2	1.6	82	2.5	2.5	1.5	1.4	2.5	9.1	1.7	1.6	4.0

Table S6 *(continued)*

Congener Group		LOQ	RD-1	RD-2	RD-3	CC-1	CC-2	CL	ASM-1	ASM-2
C10	Cl4	10	<10	<10	<10	<10	<10	6400	<10	<10
	Cl5	1.0	<1.0	<1.0	1.8	2.0	<1.0	13000	<1.0	2.4
	Cl6	1.0	5.6	4.9	4.9	<1.0	<1.0	12000	3.2	5.1
	Cl7	1.0	<1.0	<1.0	<1.0	<1.0	<1.0	4100	<1.0	<1.0
	Cl8	1.0	<1.0	<1.0	<1.0	<1.0	<1.0	620	<1.0	<1.0
C11	Cl4	5.0	<5.0	<5.0	<5.0	<5.0	<5.0	3700	<5.0	<5.0
	Cl5	1.0	<1.0	<1.0	<1.0	<1.0	<1.0	11000	<1.0	<1.0
	Cl6	1.0	<1.0	<1.0	<1.0	<1.0	<1.0	11000	<1.0	<1.0
	Cl7	1.0	<1.0	<1.0	<1.0	<1.0	<1.0	5900	<1.0	<1.0
	Cl8	1.0	<1.0	<1.0	<1.0	<1.0	<1.0	1900	<1.0	<1.0
	Cl9	1.0	<1.0	<1.0	<1.0	<1.0	<1.0	240	1.6	1.4
C12	Cl4	2.0	<2.0	<2.0	<2.0	<2.0	<2.0	2700	<2.0	<2.0
	Cl5	1.0	<1.0	<1.0	<1.0	<1.0	<1.0	7600	<1.0	<1.0
	Cl6	1.0	<1.0	<1.0	<1.0	<1.0	<1.0	9100	<1.0	<1.0
	Cl7	1.0	1.8	<1.0	1.3	<1.0	<1.0	6600	1.7	<1.0
	Cl8	1.0	<1.0	<1.0	<1.0	<1.0	<1.0	3700	19	12
	Cl9	1.0	<1.0	<1.0	<1.0	<1.0	<1.0	990	25	15
	Cl10	1.0	<1.0	<1.0	<1.0	<1.0	<1.0	100	6.6	3.9
C13	Cl4	1.0	<1.0	<1.0	<1.0	<1.0	<1.0	1700	<1.0	<1.0
	Cl5	1.0	<1.0	<1.0	<1.0	<1.0	<1.0	4700	<1.0	<1.0
	Cl6	1.0	<1.0	<1.0	<1.0	<1.0	<1.0	6400	<1.0	<1.0
	Cl7	1.0	<1.0	<1.0	<1.0	<1.0	<1.0	7800	<1.0	<1.0
	Cl8	1.0	<1.0	<1.0	<1.0	<1.0	<1.0	5800	<1.0	<1.0
	Cl9	1.0	<1.0	<1.0	<1.0	<1.0	<1.0	2100	<1.0	<1.0
	Cl10	1.0	<1.0	<1.0	<1.0	<1.0	<1.0	340	<1.0	<1.0
Summary	ΣC10		5.6	4.9	6.7	2.0	<LOQ	36000	3.2	7.5
	ΣC11		<LOQ	<LOQ	<LOQ	<LOQ	<LOQ	34000	1.6	1.4
	ΣC12		1.8	<LOQ	1.3	<LOQ	<LOQ	31000	52	31
	ΣC13		<LOQ	<LOQ	<LOQ	<LOQ	<LOQ	29000	<LOQ	<LOQ
	ΣSCCP		7.4	4.9	8.0	2.0	<LOQ	130000	57	40

Sample IDs are colour coded such that black=PVC, blue=rubber and green=unknown composition.

Table S7. Concentrations of medium-chain chlorinated paraffins (MCCPs) in polymer samples (μg/g).

Congener Group		LOQ	YM-1	YM-2	BB-1	BB-2	PM-1	PM-2	PM-3	PM-4	CH	JR-1	JR-2	JR-3	JR-4	EC-1	EC-2	FF-1	FF-2	FF-3	FF-4	FF-5
	Cl4	1.0	<1.0	<1.0	<1.0	<1.0	<1.0	<1.0	<1.0	<1.0	<1.0	<1.0	<1.0	<1.0	<1.0	<1.0	<1.0	<1.0	<1.0	<1.0	<1.0	<1.0
	Cl5	1.0	<1.0	<1.0	<1.0	<1.0	2.4	<1.0	<1.0	<1.0	<1.0	<1.0	3.0	<1.0	<1.0	<1.0	<1.0	<1.0	<1.0	<1.0	<1.0	<1.0
	Cl6	1.0	<1.0	<1.0	<1.0	<1.0	2.8	<1.0	<1.0	<1.0	<1.0	<1.0	3.7	<1.0	<1.0	<1.0	<1.0	<1.0	<1.0	<1.0	<1.0	<1.0
C14	Cl7	1.0	<1.0	<1.0	<1.0	<1.0	1.8	<1.0	<1.0	<1.0	<1.0	<1.0	2.3	<1.0	<1.0	<1.0	<1.0	<1.0	<1.0	<1.0	<1.0	<1.0
	Cl8	1.0	<1.0	<1.0	<1.0	<1.0	<1.0	<1.0	<1.0	<1.0	<1.0	<1.0	<1.0	<1.0	<1.0	<1.0	<1.0	<1.0	<1.0	<1.0	<1.0	<1.0
	Cl9	1.0	<1.0	<1.0	<1.0	<1.0	<1.0	<1.0	<1.0	<1.0	<1.0	<1.0	<1.0	<1.0	<1.0	<1.0	<1.0	<1.0	<1.0	<1.0	<1.0	<1.0
	Cl10	1.0	<1.0	<1.0	<1.0	<1.0	<1.0	<1.0	<1.0	<1.0	<1.0	<1.0	<1.0	<1.0	<1.0	<1.0	<1.0	<1.0	<1.0	<1.0	<1.0	<1.0
	Cl4	1.0	<1.0	<1.0	<1.0	<1.0	<1.0	<1.0	<1.0	<1.0	<1.0	<1.0	<1.0	<1.0	<1.0	<1.0	<1.0	<1.0	<1.0	<1.0	<1.0	<1.0
	Cl5	1.0	<1.0	<1.0	<1.0	<1.0	1.7	<1.0	<1.0	<1.0	<1.0	<1.0	1.4	<1.0	<1.0	<1.0	<1.0	<1.0	<1.0	<1.0	<1.0	<1.0
	Cl6	1.0	<1.0	<1.0	<1.0	<1.0	2.3	<1.0	<1.0	<1.0	<1.0	<1.0	1.6	<1.0	<1.0	<1.0	<1.0	<1.0	<1.0	<1.0	<1.0	<1.0
C15	Cl7	1.0	<1.0	<1.0	<1.0	<1.0	1.1	<1.0	<1.0	<1.0	<1.0	<1.0	<1.0	<1.0	<1.0	<1.0	<1.0	<1.0	<1.0	<1.0	<1.0	<1.0
	Cl8	1.0	<1.0	<1.0	<1.0	<1.0	<1.0	<1.0	<1.0	<1.0	<1.0	<1.0	<1.0	<1.0	<1.0	<1.0	<1.0	<1.0	<1.0	<1.0	<1.0	<1.0
	Cl9	1.0	<1.0	<1.0	<1.0	<1.0	<1.0	<1.0	<1.0	<1.0	<1.0	<1.0	<1.0	<1.0	<1.0	<1.0	<1.0	<1.0	<1.0	<1.0	<1.0	<1.0
	Cl10	1.0	<1.0	<1.0	<1.0	<1.0	<1.0	<1.0	<1.0	<1.0	<1.0	<1.0	<1.0	<1.0	<1.0	<1.0	<1.0	<1.0	<1.0	<1.0	<1.0	<1.0
	Cl4	1.0	<1.0	<1.0	<1.0	<1.0	<1.0	<1.0	<1.0	<1.0	<1.0	<1.0	<1.0	<1.0	<1.0	<1.0	<1.0	<1.0	<1.0	<1.0	<1.0	<1.0
	Cl5	1.0	<1.0	<1.0	<1.0	<1.0	<1.0	<1.0	<1.0	<1.0	<1.0	<1.0	<1.0	<1.0	<1.0	<1.0	<1.0	<1.0	<1.0	<1.0	<1.0	<1.0
	Cl6	1.0	<1.0	<1.0	<1.0	<1.0	1.1	<1.0	<1.0	<1.0	<1.0	<1.0	1.0	<1.0	<1.0	<1.0	<1.0	<1.0	<1.0	<1.0	<1.0	<1.0
C16	Cl7	1.0	<1.0	<1.0	<1.0	<1.0	1.1	<1.0	<1.0	<1.0	<1.0	<1.0	1.1	<1.0	<1.0	<1.0	<1.0	<1.0	<1.0	<1.0	<1.0	<1.0
	Cl8	1.0	<1.0	<1.0	<1.0	<1.0	<1.0	<1.0	<1.0	<1.0	<1.0	<1.0	<1.0	<1.0	<1.0	<1.0	<1.0	<1.0	<1.0	<1.0	<1.0	<1.0
	Cl9	1.0	<1.0	<1.0	<1.0	<1.0	<1.0	<1.0	<1.0	<1.0	<1.0	<1.0	<1.0	<1.0	<1.0	<1.0	<1.0	<1.0	<1.0	<1.0	<1.0	<1.0
	Cl10	1.0	<1.0	<1.0	<1.0	<1.0	<1.0	<1.0	<1.0	<1.0	<1.0	<1.0	<1.0	<1.0	<1.0	<1.0	<1.0	<1.0	<1.0	<1.0	<1.0	<1.0
	Cl4	1.0	<1.0	<1.0	<1.0	<1.0	<1.0	<1.0	<1.0	<1.0	<1.0	<1.0	<1.0	<1.0	<1.0	<1.0	<1.0	<1.0	<1.0	<1.0	<1.0	<1.0
	Cl5	1.0	<1.0	<1.0	<1.0	<1.0	<1.0	<1.0	<1.0	<1.0	<1.0	<1.0	<1.0	<1.0	<1.0	<1.0	<1.0	<1.0	<1.0	<1.0	<1.0	<1.0
	Cl6	1.0	<1.0	<1.0	<1.0	<1.0	<1.0	<1.0	<1.0	<1.0	<1.0	<1.0	<1.0	<1.0	<1.0	<1.0	<1.0	<1.0	<1.0	<1.0	<1.0	<1.0
C17	Cl7	1.0	<1.0	<1.0	<1.0	<1.0	<1.0	<1.0	<1.0	<1.0	<1.0	<1.0	<1.0	<1.0	<1.0	<1.0	<1.0	<1.0	<1.0	<1.0	<1.0	<1.0
	Cl8	1.0	<1.0	<1.0	<1.0	<1.0	<1.0	<1.0	<1.0	<1.0	<1.0	<1.0	<1.0	<1.0	<1.0	<1.0	<1.0	<1.0	<1.0	<1.0	<1.0	<1.0
	Cl9	1.0	<1.0	<1.0	<1.0	<1.0	<1.0	<1.0	<1.0	<1.0	<1.0	<1.0	<1.0	<1.0	<1.0	<1.0	<1.0	<1.0	<1.0	<1.0	<1.0	<1.0
	Cl10	1.0	<1.0	<1.0	<1.0	<1.0	<1.0	<1.0	<1.0	<1.0	<1.0	<1.0	<1.0	<1.0	<1.0	<1.0	<1.0	<1.0	<1.0	<1.0	<1.0	<1.0
	ΣC14		<LOQ	<LOQ	<LOQ	<LOQ	7.0	<LOQ	<LOQ	<LOQ	<LOQ	<LOQ	9.0	<LOQ	<LOQ	<LOQ	<LOQ	<LOQ	<LOQ	<LOQ	<LOQ	<LOQ
	ΣC15		<LOQ	<LOQ	<LOQ	<LOQ	5.1	<LOQ	<LOQ	<LOQ	<LOQ	<LOQ	3.0	<LOQ	<LOQ	<LOQ	<LOQ	<LOQ	<LOQ	<LOQ	<LOQ	<LOQ
Summary	ΣC16		<LOQ	<LOQ	<LOQ	<LOQ	2.2	<LOQ	<LOQ	<LOQ	<LOQ	<LOQ	2.1	<LOQ	<LOQ	<LOQ	<LOQ	<LOQ	<LOQ	<LOQ	<LOQ	<LOQ
	ΣC17		<LOQ	<LOQ	<LOQ	<LOQ	<LOQ	<LOQ	<LOQ	<LOQ	<LOQ	<LOQ	<LOQ	<LOQ	<LOQ	<LOQ	<LOQ	<LOQ	<LOQ	<LOQ	<LOQ	<LOQ
	ΣMCCP		<LOQ	<LOQ	<LOQ	<LOQ	14	<LOQ	<LOQ	<LOQ	<LOQ	<LOQ	14	<LOQ	<LOQ	<LOQ	<LOQ	<LOQ	<LOQ	<LOQ	<LOQ	<LOQ

Table S7 *(continued)*

Congener Group		LOQ	RD-1	RD-2	RD-3	CC-1	CC-2	CL	ASM-1	ASM-2
C14	Cl4	1.0	12	<1.0	<1.0	<1.0	<1.0	2.8	<1.0	<1.0
	Cl5	1.0	34	<1.0	1.2	<1.0	<1.0	60	<1.0	<1.0
	Cl6	1.0	43	<1.0	1.6	<1.0	<1.0	210	<1.0	<1.0
	Cl7	1.0	27	<1.0	2.0	<1.0	<1.0	280	<1.0	<1.0
	Cl8	1.0	11	<1.0	1.0	<1.0	<1.0	280	<1.0	<1.0
	Cl9	1.0	1.7	<1.0	<1.0	<1.0	<1.0	160	<1.0	<1.0
	Cl10	1.0	<1.0	<1.0	<1.0	<1.0	<1.0	30	<1.0	<1.0
C15	Cl4	1.0	6.3	<1.0	<1.0	<1.0	<1.0	<1.0	<1.0	<1.0
	Cl5	1.0	20	<1.0	<1.0	<1.0	<1.0	25	<1.0	<1.0
	Cl6	1.0	30	<1.0	<1.0	<1.0	<1.0	120	<1.0	<1.0
	Cl7	1.0	17	<1.0	<1.0	<1.0	<1.0	230	<1.0	<1.0
	Cl8	1.0	7.8	<1.0	<1.0	<1.0	<1.0	290	<1.0	<1.0
	Cl9	1.0	2.2	<1.0	<1.0	<1.0	<1.0	240	<1.0	<1.0
	Cl10	1.0	<1.0	<1.0	<1.0	<1.0	<1.0	88	<1.0	<1.0
C16	Cl4	1.0	6.7	<1.0	<1.0	<1.0	<1.0	<1.0	<1.0	<1.0
	Cl5	1.0	12	<1.0	<1.0	<1.0	<1.0	12	<1.0	<1.0
	Cl6	1.0	16	<1.0	<1.0	<1.0	<1.0	64	<1.0	<1.0
	Cl7	1.0	21	<1.0	<1.0	<1.0	<1.0	140	<1.0	<1.0
	Cl8	1.0	17	<1.0	<1.0	<1.0	<1.0	250	<1.0	<1.0
	Cl9	1.0	5.4	<1.0	<1.0	<1.0	<1.0	270	<1.0	<1.0
	Cl10	1.0	1.1	<1.0	<1.0	<1.0	<1.0	150	<1.0	<1.0
C17	Cl4	1.0	1.2	<1.0	<1.0	<1.0	<1.0	<1.0	<1.0	<1.0
	Cl5	1.0	4.7	<1.0	<1.0	<1.0	<1.0	6.7	<1.0	<1.0
	Cl6	1.0	12	<1.0	<1.0	<1.0	<1.0	31	<1.0	<1.0
	Cl7	1.0	17	<1.0	<1.0	<1.0	<1.0	79	<1.0	<1.0
	Cl8	1.0	13	<1.0	<1.0	<1.0	<1.0	170	<1.0	<1.0
	Cl9	1.0	4.9	<1.0	<1.0	<1.0	<1.0	220	<1.0	<1.0
	Cl10	1.0	<1.0	<1.0	<1.0	<1.0	<1.0	130	<1.0	<1.0
Summary	ΣC14		130	<LOQ	5.8	<LOQ	<LOQ	1000	<LOQ	<LOQ
	ΣC15		83	<LOQ	<LOQ	<LOQ	<LOQ	990	<LOQ	<LOQ
	ΣC16		79	<LOQ	<LOQ	<LOQ	<LOQ	890	<LOQ	<LOQ
	ΣC17		53	<LOQ	<LOQ	<LOQ	<LOQ	640	<LOQ	<LOQ
	ΣMCCP		350	<LOQ	5.8	<LOQ	<LOQ	3500	<LOQ	<LOQ

Sample IDs are colour coded such that black=PVC, blue=rubber and green=unknown composition.

Table S8. Plasticizers detected in consumer products and toys by GC-EI/MS.

Sample ID	Polymer	Plasticizers	Plasticizer Identification	Other compounds (NIST)
YM-1	PVC	DEHT	Analytical Standard	
YM-2	PVC	DEHT	Analytical Standard	
BB-1	PVC	DINCH	Analytical Standard	fatty acids C14, CA6, C18:0, C18:1
BB-2	PVC	DEHT	Analytical Standard	
PM-1	PVC	DEHT	Analytical Standard	
PM-2	PVC	DEHT	Analytical Standard	
PM-3	PVC	DEHT	Analytical Standard	
PM-4	PVC	DEHT	Analytical Standard	
CH	PVC	DEHT	Analytical Standard	
JR-1	PVC	DEHT	Analytical Standard	
JR-2	PVC	DEHT	Analytical Standard	
JR-3	PVC	DINCH	Analytical Standard	
JR-4	PVC	DINCH	Analytical Standard	
EC-1	PVC	DPHP	Analytical Standard	decyl phthalate
EC-2	PVC	DPHP	Analytical Standard	decyl phthalate
FF-1A	PVC	DEHT	Analytical Standard	
FF-2A	PVC	BTBC	NIST Match	butyl citrate
FF-3A	PVC	BTBC	NIST Match	
FF-4A	PVC	BTBC	NIST Match	
FF-5A	Rubber	DEHT	Analytical Standard	
RD-1	Rubber	DINCH+DEHT	Analytical Standard	
RD-2	Rubber	DINCH+DEHT	Analytical Standard	
RD-3	Rubber	DEHT	Analytical Standard	
CC-1	Rubber			BHT
CC-2	Rubber			
CL	Unknown	DNBP	Analytical Standard	
ASM-1	Unknown	DEHT	Analytical Standard	
ASM-2	Unknown	DEHT	Analytical Standard	

NIST = National Institute of Standards and Technology mass spectral library. PVC = polyvinyl chloride, DEHT = di-2-ethylhexyl-terephthalate, DINCH = 1,2-cyclohexane dicarboxylic acid diisononyl ester, DPHP = di-(2-propyl heptyl) phthalate, BTBC = butyryltributyl citrate, DNBP = phthalic acid esters di-n-butyl phthalate and BHT = butylated hydroxy toluene.

References

H. Matsukami, H. Takemori, T. Takasuga, H. Kuramochi, N. Kajiwara, Liquid chromatography–electrospray ionization-tandem mass spectrometry for the determination of short-chain chlorinated paraffins in mixed plastic wastes, Chemosphere, 244 (2020) 125531.
